# Subtype selective fluorescent ligands based on ICI 118,551 to study the human β2‐adrenoceptor in CRISPR/Cas9 genome‐edited HEK293T cells at low expression levels

**DOI:** 10.1002/prp2.779

**Published:** 2021-05-18

**Authors:** Joëlle Goulding, Sarah J. Mistry, Mark Soave, Jeanette Woolard, Stephen J. Briddon, Carl W. White, Barrie Kellam, Stephen J. Hill

**Affiliations:** ^1^ Division of Physiology, Pharmacology and Neuroscience, School of Life Sciences University of Nottingham Nottingham UK; ^2^ Centre of Membrane Proteins and Receptors (COMPARE) University of Birmingham and University of Nottingham Midlands UK; ^3^ School of Pharmacy University of Nottingham Nottingham UK; ^4^ Harry Perkins Institute of Medical Research and Centre for Medical Research University of Western Australia QEII Medical Centre Nedlands Western Australia Australia; ^5^ Australian Research Council Centre for Personalised Therapeutics Technologies Australia

**Keywords:** CRISPR/Cas9 genome editing, fluorescent ligands, ICI 118,551, ligand binding, NanoBRET, β_2_‐adrenoceptors

## Abstract

Fluorescent ligand technologies have proved to be powerful tools to improve our understanding of ligand‐receptor interactions. Here we have characterized a small focused library of nine fluorescent ligands based on the highly selective β_2_‐adrenoceptor (β_2_AR) antagonist ICI 118,551. The majority of fluorescent ICI 118,551 analogs had good affinity for the β_2_AR (pK_D_ >7.0) with good selectivity over the β_1_AR (pK_D_ <6.0). The most potent and selective ligands being **8c** (ICI 118,551‐Gly‐Ala‐BODIPY‐FL‐X; β_2_AR pK_D_ 7.48), **9c** (ICI 118,551‐βAla‐βAla‐BODIPY‐FL‐X; β_2_AR pK_D_ 7.48), **12a** (ICI 118,551‐PEG‐BODIPY‐X‐630/650; β_2_AR pK_D_ 7.56), and **12b** (ICI 118,551‐PEG‐BODIPY‐FL; β_2_AR pK_D_ 7.42). **9a** (ICI 118,551‐βAla‐βAla‐BODIPY‐X‐630/650) had the highest affinity at recombinant β_2_ARs (pK_D_ 7.57), but also exhibited significant binding affinity to the β_1_AR (pK_D_ 6.69). Nevertheless, among the red fluorescent ligands, **9a** had the best imaging characteristics in recombinant HEK293 T cells and labeling was mostly confined to the cell surface. In contrast, **12a** showed the highest propensity to label intracellular β_2_ARs in HEK293 T cell expressing exogenous β_2_ARs. This suggests that a combination of the polyethylene glycol (PEG) linker and the BODIPY‐X‐630/650 makes this ICI 118,551 derivative particularly susceptible to crossing the cell membrane to access the intracellular β_2_ARs. We have also used these ligands in combination with CRISPR/Cas9 genome‐edited HEK293 T cells to undertake for the first time real‐time ligand binding to native HEK293 T β_2_ARs at low native receptor expression levels. These studies provided quantitative data on ligand‐binding characteristics but also allowed real‐time visualization of the ligand‐binding interactions in genome‐edited cells using NanoBRET luminescence imaging.

## Graphical abstract

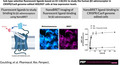

## 1 INTRODUCTION

Beta‐adrenoceptors (βARs) are Class A G protein‐coupled receptors (GPCRs) which have been successfully targeted for the treatment of cardiovascular and respiratory disorders.^1^, ^2^ There are three receptor subtypes, β_1_AR, β_2_AR, and β_3_AR, which display tissue‐specific localization and functionality.^3^, ^4^, ^5^ All three subtypes are found in the heart, with the β_1_AR being the most abundant and is able to regulate heart rate and contractile force.^6^ β_2_ARs can couple to both G_αs_ and G_αi_ heterotrimeric G proteins.^7^, ^8^, ^9^ They are found within the smooth muscle of the airways and can be targeted to relieve symptoms of respiratory disorders, such as asthma and chronic obstructive pulmonary disease.^2^, ^9^ The β_3_AR has limited expression, chiefly in adipose tissue and the bladder, but can also be found at low expression levels in the heart.^10^, ^11^, ^12^

The β_2_AR is a commonly used model for Class A GPCRs within research. This has been facilitated by the myriad of tools with which to study its role and function, including numerous published crystal structures showing the receptor in an inactive state bound to the negative allosteric nanobody 60,^13^ as well as the agonist,^14^, ^15^, ^16^ inverse agonist,^17^ and G protein‐bound states.^15^ Traditionally, ligand‐binding parameters have been determined using radiolabeled ligands as the pharmacological probe. However, these approaches are not readily applicable to the study of receptor properties in living cells and in real time.

Recent advances in fluorescent ligand design have allowed the development of live‐cell real‐time ligand‐receptor binding assays.^18^, ^19^, ^20^ Fluorescent ligands can inform on receptor localization, expression level, ligand affinity, receptor specificity, and ligand‐binding kinetics through the use of imaging techniques, and those based on resonance energy transfer.^18^, ^19^, ^20^, ^21^, ^22^, ^23^, ^24^ The composition of the fluorescent ligand (the orthosteric binding moiety, linker and fluorophore) can exert significant effects on its utility as a probe, including modulating the selectivity profile of the probe and changing its photochemical properties, which affects its use for confocal microscopy.^25^ To date, there are no highly selective fluorescent probes available to study the β_2_AR, and many existing βAR fluorescent ligands have very limited β_2_AR/β_1_AR selectivity.^26^, ^27^

Fusion of a luciferase to a receptor of interest has allowed a range of biological effects to be investigated using bioluminescence resonance energy transfer (BRET).^28^ We have recently shown how fluorescent ligands can be used in combination with nanoluciferase to provide an exquisitely sensitive NanoBRET ligand‐binding assay.^18^, ^21^, ^29^ Until recently, however, the use of NanoBRET to investigate ligand‐binding properties required the exogenous expression of the nanoluciferase‐tagged GPCR of interest, and this can affect how receptors interact and signal. To maintain the normal cellular context, we have previously used CRISPR/Cas9‐mediated homology‐directed repair to insert luminescent tags into the endogenous genome.^30^, ^31^, ^32^ Here, we describe novel and highly selective fluorescent ligands based on the β_2_AR selective antagonist ICI 118,551^33^ and linked to different fluorophores with a variety of linkers. We characterize these ligands in terms of βAR affinity and selectivity using a NanoBRET proximity‐based binding assay^29^ and examine their imaging properties via confocal microscopy. We also use them to demonstrate fluorescent ligand binding to genome‐edited β_2_ARs at low native levels of receptor expression in HEK293 T cells.

## 2 MATERIALS AND METHODS

### 2.1 Materials

FuGENE and furimazine were obtained from Promega. SNAP‐Surface®, Alexa Fluor® 488 and Alexa Fluor 647 were obtained from New England Biolabs. ^3^H‐CGP12177 and MicroScint 20 were from Perkin Elmer. All other chemicals were from Sigma‐Aldrich. Nunc™ Lab‐Tek™ chambered coverglass (155361) was obtained from Thermo Fisher Scientific. 96‐well white clear‐bottomed plates and 35 mm Cellview 4‐quadrant culture dishes were from Greiner Bio‐One.

### 2.2 Chemistry

Chemicals and solvents of analytical and HPLC grade were purchased from commercial suppliers and used without further purification. BODIPY‐630/650‐X‐SE, BODIPY‐FL‐X‐SE, and BODIPY‐FL‐SE were purchased from Molecular Probes (Thermo Fisher Scientific). All reactions were carried out at ambient temperature unless otherwise stated. Reactions were monitored by thin‐layer chromatography on commercially available silica pre‐coated aluminum‐backed plates (Merck Kieselgel 60 F254). Visualization was under UV light (254 nm and 366 nm), followed by staining with ninhydrin or KMnO_4_ dips. Flash column chromatography was performed using silica gel 60, 230–400 mesh particle size (Sigma‐Aldrich). NMR spectra were recorded on a Bruker‐AV 400. ^1^H spectra were recorded at 400.13 Hz and ^13^C NMR spectra at 101.62 Hz. All ^13^C NMR are ^1^H broadband decoupled. Solvents used for NMR analysis (reference peaks listed) were CDCl_3_ supplied by Cambridge Isotope Laboratories Inc., (δ_H_ = 7.26 ppm, δ_C_ = 77.16) and CD_3_OD supplied by VWR (δ_H_ = 3.31 ppm and δ_C_ = 49.00). Chemical shifts (δ) are recorded in parts per million (ppm) and coupling constants are recorded in Hz. The following abbreviations are used to describe signal shapes and multiplicities; singlet (s), doublet (d), triplet (t), quadruplet (q), broad (br), dd (doublet of doublets), ddd (double doublet of doublets), dtd (double triplet of doublets), and multiplet (m). Spectra were assigned using appropriate COSY and HSQC experiments. Processing of the NMR data was carried out using the NMR software Topspin 3.0. LC‐MS spectra were recorded on a Shimadzu UFLCXR system coupled to an Applied Biosystems API2000 and visualized at 254 nm (channel 1) and 220 nm (channel 2). LC‐MS was carried out using a Phenomenex Gemini‐NX C18 110A, column 50 mm × 2 mm x 3 μm at a flow rate of 0.5 ml/min over a 5 min period. All high‐resolution mass spectra (HRMS) were recorded on a Bruker microTOF mass spectrometer using MS electrospray ionization operating in positive ion mode. RP‐HPLC was performed on a Waters 515 LC system and monitored using a Waters 996 photodiode array detector at wavelengths between 190 and 800 nm. Spectra were analyzed using Millennium 32 software. Semi‐preparative HPLC was performed using YMC‐Pack C8 column (150 mm ×10 mm ×5 μm) at a flow rate of 5.0 ml/min using a gradient method of 40–95% B over 15 minutes (solvent A = 0.01% formic acid in H_2_O, solvent B = 0.01% formic acid in CH_3_CN (method A)) or 40–75% B over 10 minutes (solvent A = 0.01% formic acid in H_2_O, solvent B = 0.01% formic acid in CH_3_CN (method B)). Analytical RP‐HPLC was performed using a YMC‐Pack C8 column (150 mm ×4.6 mm ×5 μm) at a flow rate of 1.0 ml/min. Final products were one single peak and >95% pure. The retention time of the final product is reported using a gradient method of 5–95% solvent B in solvent A over 25 minutes (solvent A = 0.01% formic acid in H_2_O, solvent B = 0.01% formic acid in CH_3_CN).

Full experimental detail for the synthesis of fluorescent ligands can be found in the Data S1.

### 2.3 Cell lines

HEK cell lines were maintained in Dulbecco's Modified Eagle Medium (DMEM; Sigma‐Aldrich) supplemented with 10% fetal bovine serum (FBS; Sigma‐Aldrich) at 37°C in 5% CO_2_. The NLuc‐β_2_AR stable cell line was obtained from Promega. The NLuc‐β_1_AR^34^ was transiently expressed in HEK293 GloSensor cells (Promega) using FuGENE (Promega), according to the manufacturer's instructions. Briefly, HEK293 GloSensor cells (120, 000/well) were plated on a clear 6‐well plate in 500 µl complete medium. Twenty‐four hours after seeding, the cells were transiently transfected with 1 µg/well NLuc‐β_1_AR in a 1:3 ratio of DNA:FuGENE and incubated at 37°C in 5% CO_2_:air for a further 24 hours before being used for NanoBRET experiments. The SNAP‐β_2_AR clonal stable cell line was a gift from Dr Karolina Gherbi (University of Nottingham). CRISPR/Cas9 genome engineering of the N‐terminal region of the β_2_AR genomic locus in HEK293 T cells to incorporate either a SNAP‐ or an NLuc‐tag was performed as described previously.^32^, ^35^ Chinese Ovary Hamster (CHO) cell lines stably expressing either the β_1_AR or β_2_AR were maintained in DMEM/F12 supplemented with 10% FBS and 2 mM L‐glutamine at 37°C in 5% CO_2_. The CHO cell lines were a gift from Prof. Jillian Baker (University of Nottingham).

### 2.4 NanoBRET binding assay

Saturation and competition binding assays were performed as previously described.^29^ Cells were seeded in 96‐well white clear‐bottomed Greiner plates pre‐treated with 10 µg/ml poly‐D‐lysine (Sigma‐Aldrich) at a density of 35,000 cells per well in DMEM supplemented with 10% FBS. The following day, the media were removed and cells were incubated with the fluorescent ICI 118,551 derivative in the presence or absence of 10 μM propranolol (saturation binding assays) or competing ligand in the presence of 10 nM fluorescent ligand (competition binding assays) in HEPES‐buffered saline solution (HBSS; 145 mM NaCl, 5 mM KCl, 1.3 mM CaCl_2_, 1 mM MgSO_4_, 10 mM HEPES, 2 mM sodium pyruvate, 1.5 mM NaHCO_3_, and 10 mM D‐glucose, pH 7.45) with 0.1% bovine serum albumin for 1 hr at 37°C. The NanoLuc substrate, furimazine (Promega), was then added to each well (10 µM final concentration) and the plate was incubated for 15 min in the dark at 37°C. The resulting BRET was measured using a PHERAstar FS plate reader (BMG Labtech) at room temperature. For each well filtered light emissions at 460 nm (80 nm bandpass) and >610 nm (longpass) for the BODIPY 630/650 ligands, and at 475 nm (30 nm bandpass) and 535 nm (30 nm bandpass) for the BODIPY‐FL and BODIPY‐X‐FL ligands were simultaneously measured. BRET ratios were calculated by dividing the longer wavelength emission by the 460 nm or 475 nm emission, respectively.

### 2.5 Radioligand binding

Competition binding assays were performed as previously described^36^ using CHO‐β_1_AR or CHO‐β_2_AR cell lines. Briefly, cells, plated in 96‐well white clear‐bottomed Greiner plates, were incubated for 2 h at 37°C in 5% CO_2_ in the presence of 0.8–1.2 nM ^3^H‐CGP 12177 (Perkin Elmer) and competing ligands (10 pM – 10 µM) in serum‐free medium. Non‐specific binding was assessed by incubating in the presence of 10 µM propranolol. Cells were washed twice in PBS before addition of 200 µL MicroScint 20 (PerkinElmer), then incubated in the dark overnight before being read on a TopCount Microplate Scintillation Counter (Packard Instrument, CT).

### 2.6 Confocal imaging

SNAP‐β_2_AR cells were seeded onto poly‐D‐lysine‐coated (10 µg/ml) 8‐well Nunc™ Lab‐Tek™ chambered coverglass (No. 1.0 borosilicate glass bottom) in DMEM supplemented with 10% FBS at a density of 10–15,000 cells per well 2 days prior to experiment. On the day of the experiment, media were replaced with labeling media which contained SNAP‐Surface® Alexa Fluor® 488 or 647 (New England Biolabs) at a final concentration of 0.5 μM (for 30 min at 37ºC). Cells were then washed in warm HBSS before a final addition of 200 μl of HBSS per well.

Cells were imaged on a Zeiss LSM880 with a Zeiss Axio Observer Z1 stand (Carl Zeiss) with a 40× C‐apochromat NA1.2 water immersion objective. Excitation was via 488 nm Argon and 633 nm helium‐neon laser lines with a 488/561/633 multibeam splitter and emission collected using a 493–628 bandpass or 638–737 bandpass. The pinhole was set at 1 Airy unit for the longer wavelength and laser power and gain and offset settings kept constant within experiment to allow comparison. Cells were imaged live at 24ºC following a 30 min pre‐incubation at 37ºC in the presence of fluorescent ligand (100 nM) following a 30 min pre‐incubation at 37ºC in the presence or absence of 10 μM propranolol. Equatorial plane images were made and four images captured per condition per experiment using ZEN 2012 software (Carl Zeiss).

### 2.7 NanoBRET imaging

Cells were seeded onto 35 mm Cellview 4‐quadrant culture dishes (Greiner Bio‐one), which have a 10 mm glass coverslip bottom, in DMEM supplemented with 10% FBS at a density of 100,000 cells per quadrant 2 days prior to experiment. On the day of the experiment, media were replaced with HBSS in the presence or absence of fluorescent ligand (100 nM) and/or propranolol (10 µM) and incubated on the microscope for 30 mins at 37°C before imaging. Bioluminescence and NanoBRET imaging were performed on an Olympus LuminoView 200 microscope with a 60x NA1.42 oil immersion objective with a 0.5x tube lens, following addition of furimazine (5 µM) (Promega). Images were captured by a C9100‐23B IMAGE EMX2 camera (Hamamatsu, Japan) with gain set at 200 for all channels. Filtered bioluminescence was captured using a 438/24 nm band‐pass filter (5 s – 1 min exposure time dependent on expression level), BRET in the presence of **9a** was captured using a 650/50 nm band‐pass filter and in the presence of **12c** using a 509/22 nm band‐pass filter. For the NLuc‐β_2_AR stable cell line, exposure times were set at 10 s (**12a**,**9a**) or 20 s (**9c**) for filtered bioluminescence and 1 min (**9a, 12a**) or 20 s (**9c**) for BRET, and for the HEK293 T cells expressing NLuc‐β_2_AR under endogenous promotion, exposure times were set at 1 min for filtered bioluminescence and 4 min for BRET. Raw intensity values were determined for 3–5 regions of interest per experiment per condition and the BRET ratio was calculated by dividing the raw intensity recorded from the BRET capture by the filtered bioluminescence capture. Corrected BRET ratios were determined by subtracting the BRET ratio determined from a control quadrant (HBSS alone). A minimum of three separate experiments were performed for each condition.

### 2.8 Data analysis

Data were analyzed using Prism 7.4 software (GraphPad, San Diego, USA). Saturation NanoBRET curves were fitted simultaneously for total (fluorescent ICI 118,551 ligand) and non‐specific binding (in the presence of 10 µM propranolol) using the following equation:

$$Total\;binding\;=\;\;\frac{B_{\max}\times \left[B\right]}{\left[B\right]+K_{D}}+m\times B+c$$

where *B_max_* is the maximal specific binding, [B] is the concentration of the fluorescent ligand (nM), *K_D_* is the equilibrium dissociation constant (nM), *m* is the slope of the non‐specific binding component, and *c* is the y‐axis intercept.

The affinities of ligands at the β_2_‐AR were calculated from competition binding data with a one‐site sigmoidal response curve given by the following equation:

$$\%\;Inhibition\;of\;specific\;binding\;=\;\frac{\left(100\times \left[A^{n}\right]\right)}{\left(\left[A^{n}\right]\times {\mathrm{IC}}_{50}^{n}\right)}$$

where [A] is the concentration of unlabeled ligand, *n* is the Hill coefficient, and *IC_50­_* is the concentration of ligand required to inhibit 50% of fluorescent ligand. The *IC_50_* values were then used to calculate the K_i_ values using the Cheng‐Prusoff equation:

$$K_{i}\;=\;\frac{{\mathrm{IC}}_{50}}{1+\frac{\left[L\right]}{K_{D}}}$$

where [L] is the concentration of fluorescent ICI 118,551 ligand in nM, and *K_D_* is the dissociation constant of either the fluorescent ligand (NanoBRET assay) or ^3^H‐CGP 12177 (radioligand assay) in nM. The K_D_ of 3H‐CGP 12177 has previously been reported for the CHO cell lines employed here^36^ as 0.42 nM (β1) and 0.17 nM (β_2_).

Bioluminescence and NanoBRET images were analyzed using ImageJ (http://rsb.info.nih.gov/ij; NIH, USA) and the Time Series Analyzer version 3.0 (https://imagej.nih.gov/ij/plugins/time‐series.html).^37^

### 2.9 Nomenclature of targets and ligands

Key protein targets and ligands in this article are hyperlinked to corresponding entries in http://www.guidetopharmacology.org, the common portal for data from the IUPHAR/BPS Guide to PHARMACOLOGY,^38^ and are permanently archived in the Concise Guide to PHARMACOLOGY 2019/20.^39^

## 3 RESULTS

### 3.1 Synthesis of ICI 118,551 fluorescent ligands

**FIGURE 1:**
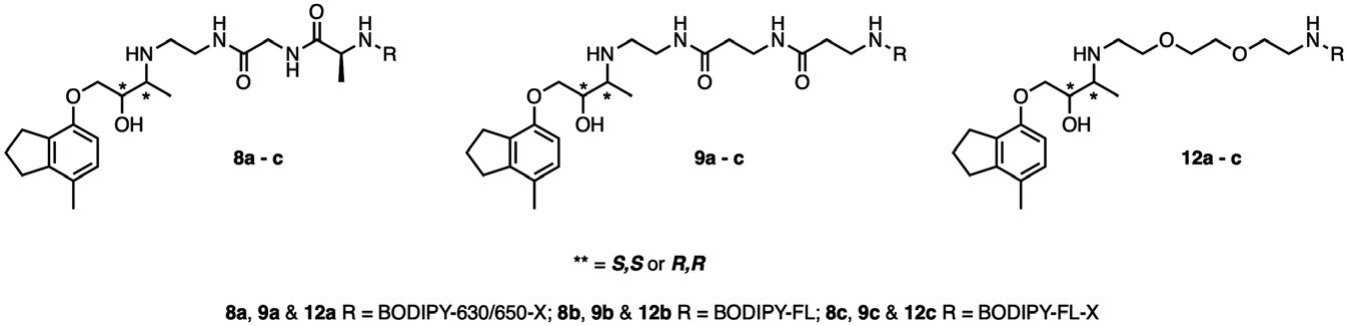
Structures of fluorescent ICI 118,551 derivatives

The highly β_2_AR‐selective antagonist ICI 118,551 was used as the starting point for the development of selective β_2_AR fluorescent ligands. The crystal structure of the human β_2_AR bound to this ligand has been previously solved (PDB 3NY8^40^ ;) and this aided identification of an appropriate attachment point on ICI 118,551 for a fluorophore via different linkers. In parallel to our own previous successes in designing and synthesizing fluorescent β‐adrenoceptor ligands,^27^, ^41^ the crystal structure suggested the alkylated amine as an ideal tether‐point, while ensuring retention of its basic pharmacophoric properties. We therefore generated a small focused library of nine ligands based on combinations of three linkers and three fluorophores (Figure 1), since previous work had demonstrated that both moieties can have a significant effect on affinity and imaging properties.^25^, ^42^, ^43^ The linkers connecting the ICI 118,551 orthostere to the fluorophore consisted of a polyethylene glycol (PEG) chain (**12a‐c**), a Gly‐Ala dipeptide linker (**8a‐c**), or a β‐Ala‐β‐Ala (**9a‐c**) linker (Figure 1), and were chosen based on earlier experience and the success of PEG and β‐Ala‐β‐Ala linkers in previous work with fluorescent propranolol derivatives [27, 41]. To allow spectral choice for these tools, BODIPY‐630/650‐X (red‐emitting), BODIPY‐FL (green‐emitting), or BODIPY‐FL‐X (green‐emitting) fluorophores were utilized. The synthetic approach to the generation of these fluorescent ligands is detailed in Data S1 and Figure S1.

### 3.2 Pharmacological characterization of fluorescent ICI 118,551 analogs

Initial NanoBRET saturation binding experiments^29^ were performed with all nine fluorescent ligands at the NLuc‐β_2_AR and NLuc‐β_1_AR receptors to determine their affinity (K_D_) for each receptor and thereby determine their β_2_/β_1_AR selectivity. The NanoBRET binding assay has been successfully implemented for ligand‐binding studies at the NLuc‐β_1_AR^34^ and NLuc‐β_2_AR^29^, ^41^ using propranolol‐based fluorescent probes. An established stable HEK293 T cell line expressing the NLuc‐β_2_AR was used for the evaluation of ligand‐binding properties at the β_2_AR, whereas β_1_AR affinities were determined in HEK293 T cells transiently transfected to express NLuc‐β_1_AR.

**FIGURE 2:**
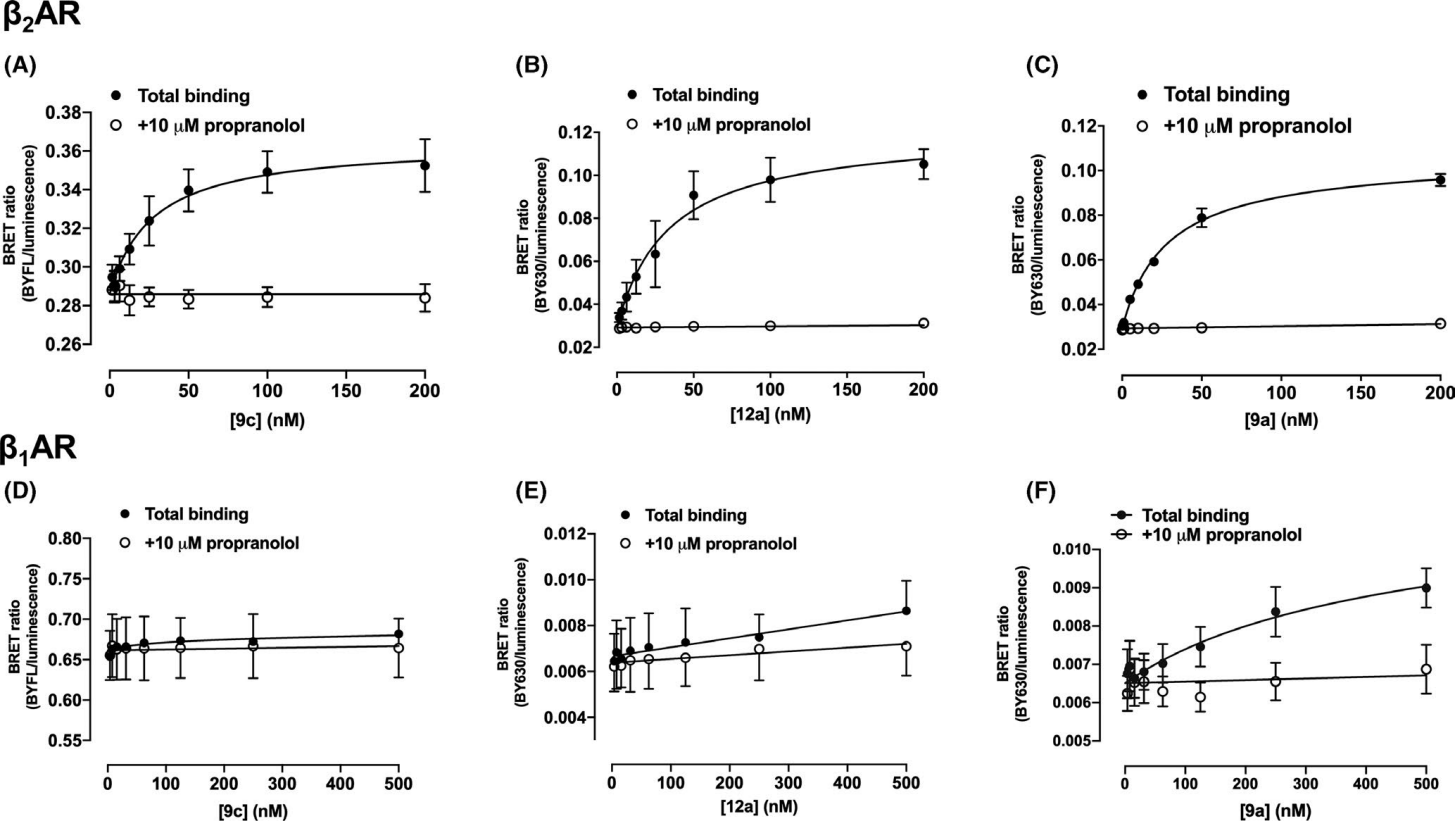
NanoBRET saturation binding curves of fluorescent ICI 118,551 derivatives in HEK293 T cells exogenously expressing NLuc‐β_2_AR or NLuc‐β_1_AR. Cells were incubated with 9c (A,D), 12a (B,E), and 9a (C,F) binding to HEK293 T cells expressing NLuc‐β_2_AR (A‐C) or NLuc‐β_1_AR (D‐F). Data were obtained in the absence (filled circles) and presence (open circles) of 10 μM propranolol. Data are mean ±S.E.M obtained in six (A), five (B, C, D, F), or four (E) separate experiments. Two‐way ANOVA (with treatments and concentrations as variables) was used to test for differences between total and non‐specific binding for each fluorescent ligand. Significant differences (*P* < .05) were obtained for the data in A, B, C, and F

All nine fluorescent ligands displayed saturable binding at the NLuc‐β_2_AR (Figure 2; Table 1). Minimal non‐specific binding was observed following co‐incubation with 10 μM propranolol. The majority of the fluorescent ligands displayed minimal specific binding at the NLuc‐β_1_AR up to the highest concentration used (500 nM) in this assay (Figure 2; Table 1). Only one compound (**9a**) displayed significant specific binding (*p*<0.05; two‐way ANOVA) to the NLuc‐β_1_AR over this concentration range (pK_D_ 6.65 ± 0.15, *n* = 6; Figure 2F). The estimated affinity for **9a** at the NLuc‐β_1_AR was, however, an order of magnitude lower than that measured at the NLuc‐β_2_AR (pK_D_ 7.57 ± 0.06, *n* = 5; Table 1).

**TABLE 1:** Dissociation constants, pK_D_, of nine fluorescent ICI 118,551 ligands for the NLuc‐β_2_AR and NLuc‐β_1_AR determined from NanoBRET saturation binding curves. Data are mean pK_D_ ± S.E.M for n separate experiments. NSB; no specific binding detected at the highest concentration of fluorescent ligand used (500 nM)

No	Fluorescent ICI 118,551 analog	β_2_AR pK_D_	*n*	β_1_AR pK_D_	*n*
8a	ICI 118,551‐Gly‐Ala‐BODIPY‐X−630/650	7.48 ± 0.08	6	NSB	5
8b	ICI 118,551‐Gly‐Ala‐BODIPY‐FL	6.31 ± 0.11	4	NSB	5
8c	ICI 118,551‐Gly‐Ala‐BODIPY‐FL‐X	7.48 ± 0.10	6	NSB	5
9a	ICI 118,551‐βAla‐βAla‐BODIPY‐X−630/650	7.57 ± 0.06	5	6.69 ± 0.15	6
9b	ICI 118,551‐βAla‐βAla‐BODIPY‐FL	7.07 ± 0.10	5	NSB	5
9c	ICI 118,551‐βAla‐βAla‐BODIPY‐FL‐X	7.48 ± 0.08	5	NSB	5
12a	ICI 118,551‐PEG‐BODIPY‐X−630/650	7.56 ± 0.08	5	NSB	5
12b	ICI 118,551‐PEG‐BODIPY‐FL	7.42 ± 0.18	4	NSB	5
12c	ICI 118,551‐PEG‐BODIPY‐FL‐X	7.25 ± 0.04	5	NSB	5

**FIGURE 3:**
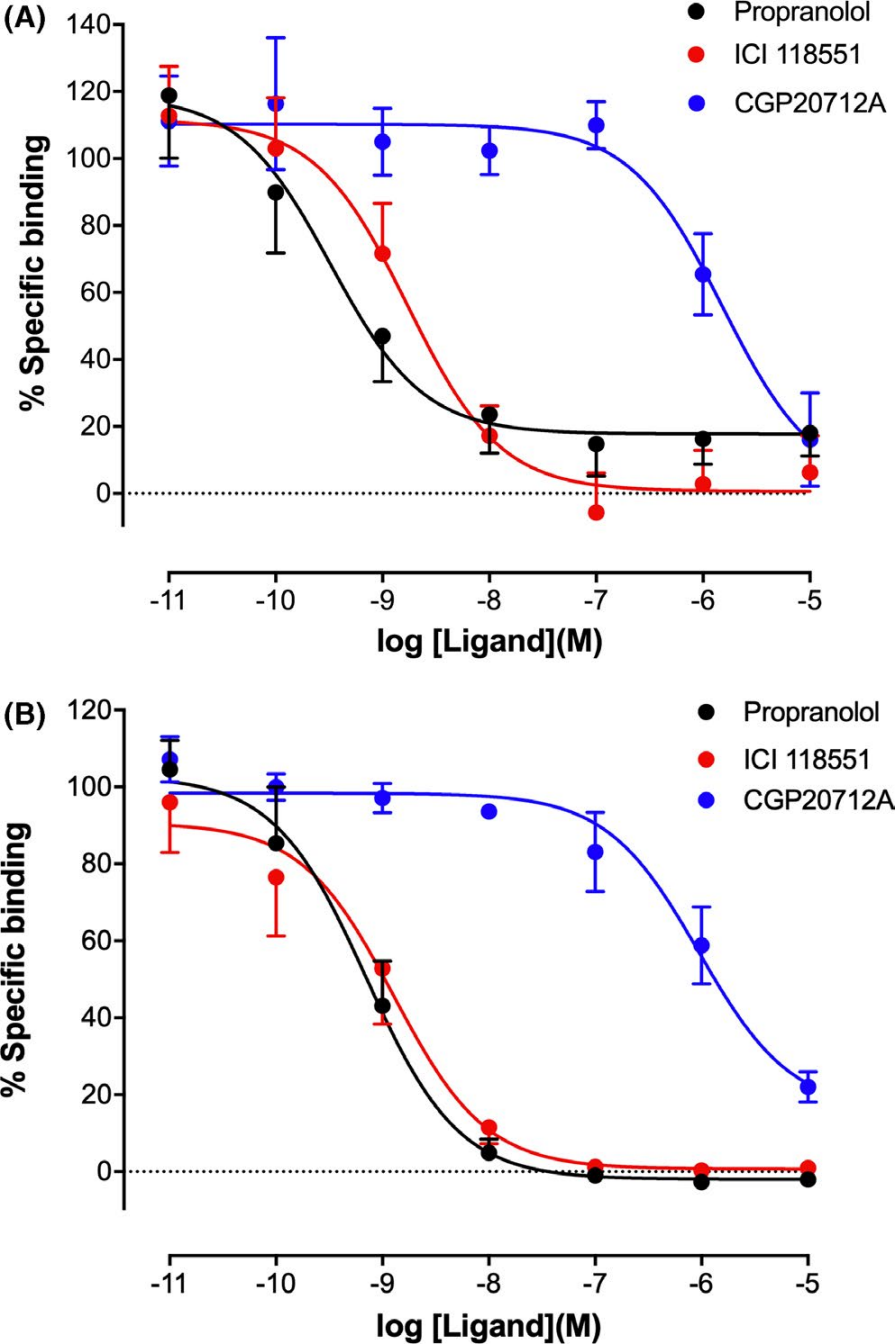
NanoBRET competition binding in HEK293 T cells exogenously expressing NLuc‐β_2_AR. Cells were incubated with 10 nM 9c (A) or 12a (B) in the absence or presence of competing ligands. Data are mean ±S.E.M. from five or six independent experiments. The actual number of repeat experiments is provided in Table 2

Two fluorescent ligands with different spectral properties were selected for further characterization. **9c** (ICI 118,551‐Gly‐Ala‐BODIPY‐FL‐X; green ligand) and **12a** (ICI 118,551‐PEG‐BODIPY‐630/650‐X; red ligand) were selected due to their β_2_AR/β_1_AR selectivity, affinity at the NLuc‐β_2_AR, and their different fluorophores, respectively. The specific binding of both ligands was inhibited by a panel of βAR ligands (Figure 3). The resulting affinities of these unlabeled ligands at the NLuc‐β_2_AR were in good agreement with literature values previously determined with radioligand binding at the untagged β_2_AR (Table 2).^36^, ^44^

**TABLE 2:** Dissociation constants, pK_i_, of unlabeled compounds obtained from inhibition of the specific binding of 10 nM 9c or 12a to NLuc‐β_2_AR. Data are mean pK_i_ ±S.E.M from n separate experiments, where each experiment was performed in triplicate

	(pK_i_)			
	9c	*n*	12a	*n*
Propranolol	9.48 ± 0.35	5	9.55 ± 0.32	6
ICI 118,551	8.95 ± 0.26	6	8.98 ± 0.21	6
CGP 20712A	6.03 ± 0.16	5	6.19 ± 0.27	6
Salmeterol	8.45 ± 0.31	6	9.01 ± 0.36	5

In order to check that the NLuc tag did not influence ligand binding to each βAR subtype, we performed competition radioligand binding studies in CHO cells stably expressing the wild‐type β_1_AR or β_2_AR subtype. CHO cells were chosen since they do not endogenously express βARs. β_2_AR/β_1_AR selectivity was maintained with **9c** and **12a** displaying comparable affinities to those seen in the NanoBRET assays (Table 3; Figure S2).

**TABLE 3:** Dissociation constants, pK_i_, determined from inhibition of specific binding of ^3^H‐CGP 12177 to β_2_AR or β_1_AR expressed in CHO cells. ND, Not determined; no inhibition of specific binding greater than 50% was observed at the maximal concentration tested (1 μM for 9c and 12a; 10 μM for CGP 20712A). Data are mean pK_i_ ±S.E.M from n separate experiments, each performed in triplicate

	(pK_i_)			
	β_2_AR	*n*	β_1_AR	*n*
9c	8.29 ± 0.17	5	ND	6
12a	7.64 ± 0.21	5	ND	6
ICI 118,551	8.76 ± 0.22	5	6.44 ± 0.14	6
CGP 20712A	ND	5	8.37 ± 0.18	6

### 3.3 Confocal imaging of β_2_AR in transfected HEK293 T cells

**FIGURE 4:**
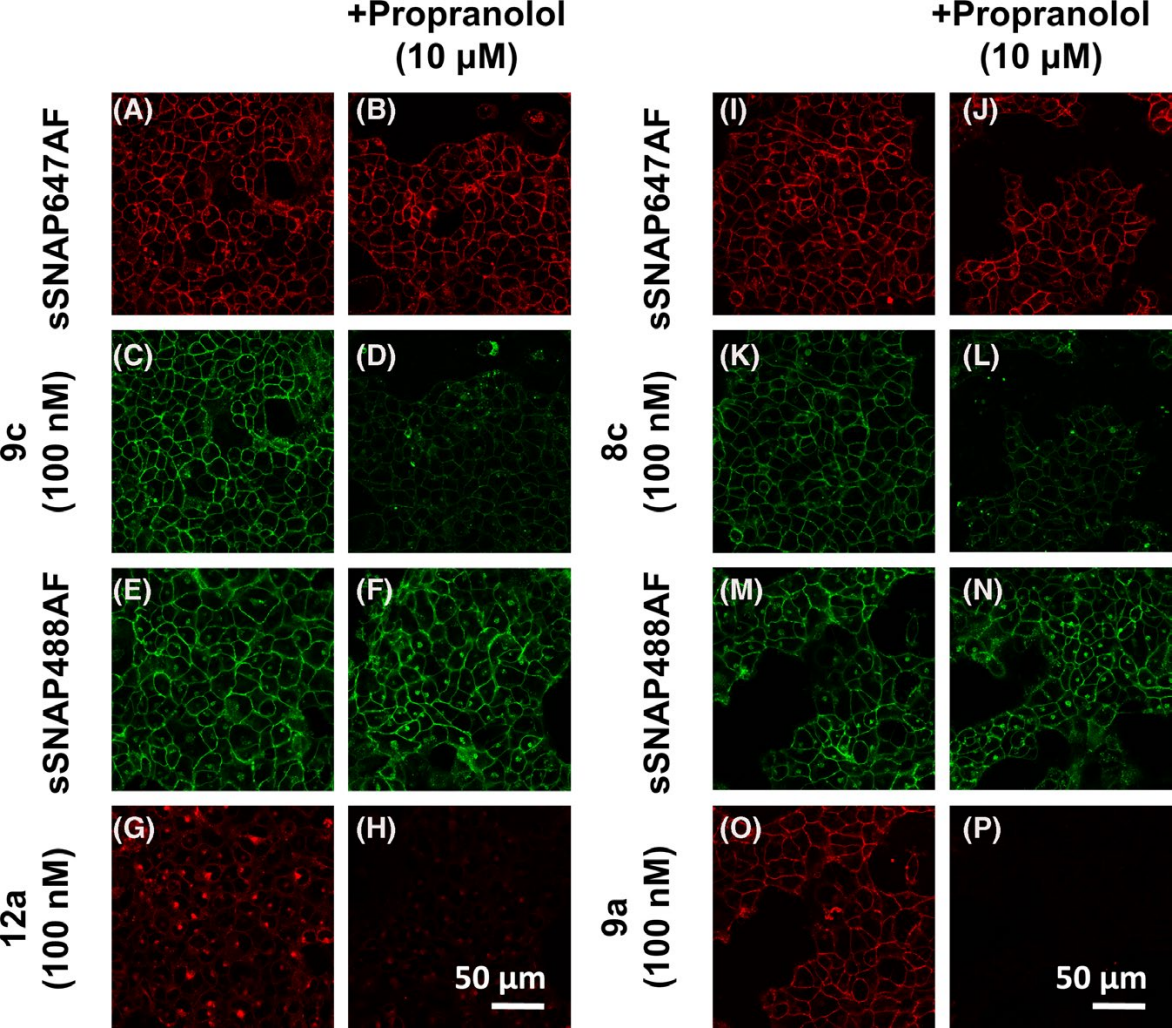
Confocal imaging of fluorescent ICI 118,551 derivatives on HEK293 T cells expressing the SNAP‐β_2_AR. Cells were incubated in fluorescent ligand (100 nM) 9c (A‐D), 12a (E‐H), 8c (I‐L), and 9a (M‐P) following 30 min incubation in the presence or absence of 10 μM propranolol. Fluorescent localization of the β_2_AR (C, G, K, O) matches that illustrated by the SNAP‐tagged dye (A, E, I, M) and is prevented from binding after propranolol addition (D, H, L, P). Similar data were obtained in three further experiments

To assess the imaging properties of two ICI 118,551 derivatives containing the green BODIPY‐FL‐X fluorophore (**9c, 8c)** and two containing the red BODIPY‐X‐630/650 fluorophore (**9a, 9c**), confocal microscopy was carried out on an HEK293 T cell line stably expressing the SNAP‐β_2_AR. The cells were labeled with SNAP surface Alexa Fluor 647 or 488, depending on the fluorescent ligand under investigation. Equatorial plane images following 30‐minute pre‐incubation of the cells with fluorescent ligand (100 nM) at 37ºC displayed clear membrane localization of the ligand which matched that observed with the SNAP‐labeled receptor (Figure 4). Some SNAP‐labeled receptor was also observed within the cytosol of cells indicating the presence of internalized receptor. The SNAP label is cell‐impermeant and as such any label internal to the cell must have been actively internalized alongside the receptor.

All four fluorescent ICI 118,551 derivatives were detected to differing extents inside cells matching the distribution of β_2_ARs seen with the SNAP dye. This suggested that the fluorescent ligand had either been internalized along with receptor or is able to cross the cell membrane independently and then bind to intracellular receptors. The presence of internalized SNAP‐β_2_AR associated with fluorescent ligand was most apparent with **12a** (Figure 4G), which suggests that a combination of the PEG linker and the BODIPY‐X‐630/650 makes this ICI 118,551 derivative particularly susceptible to crossing the cell membrane to the intracellular regions of the cell (Figure 4G).

No wash steps were performed in these experiments, but free fluorescent ligand was not detected within the imaging buffer. This is a property common to the BODIPY fluorophores, which appear to have a greater quantum yield when bound to GPCRs in a lipid environment.^45^ To establish the specific binding of **8c, 9a**, **9c,** and **12a** to the SNAP‐β_2_AR, cells were pre‐incubated with 10 μM propranolol for 30 minutes prior to addition of fluorescent ligand. A clear reduction in membrane fluorescence of all four fluorescent ligands was observed, indicating that the majority of fluorescence denotes specific binding to the SNAP‐β_2_AR (Figure 4; Figure S3). This was also true of the cytosolic receptor binding.

### 3.4 NanoBRET imaging of the binding of 9a, 9c, and 12a to transfected HEK293 T cells expressing recombinant NLuc‐β_2_ARs

**FIGURE 5:**
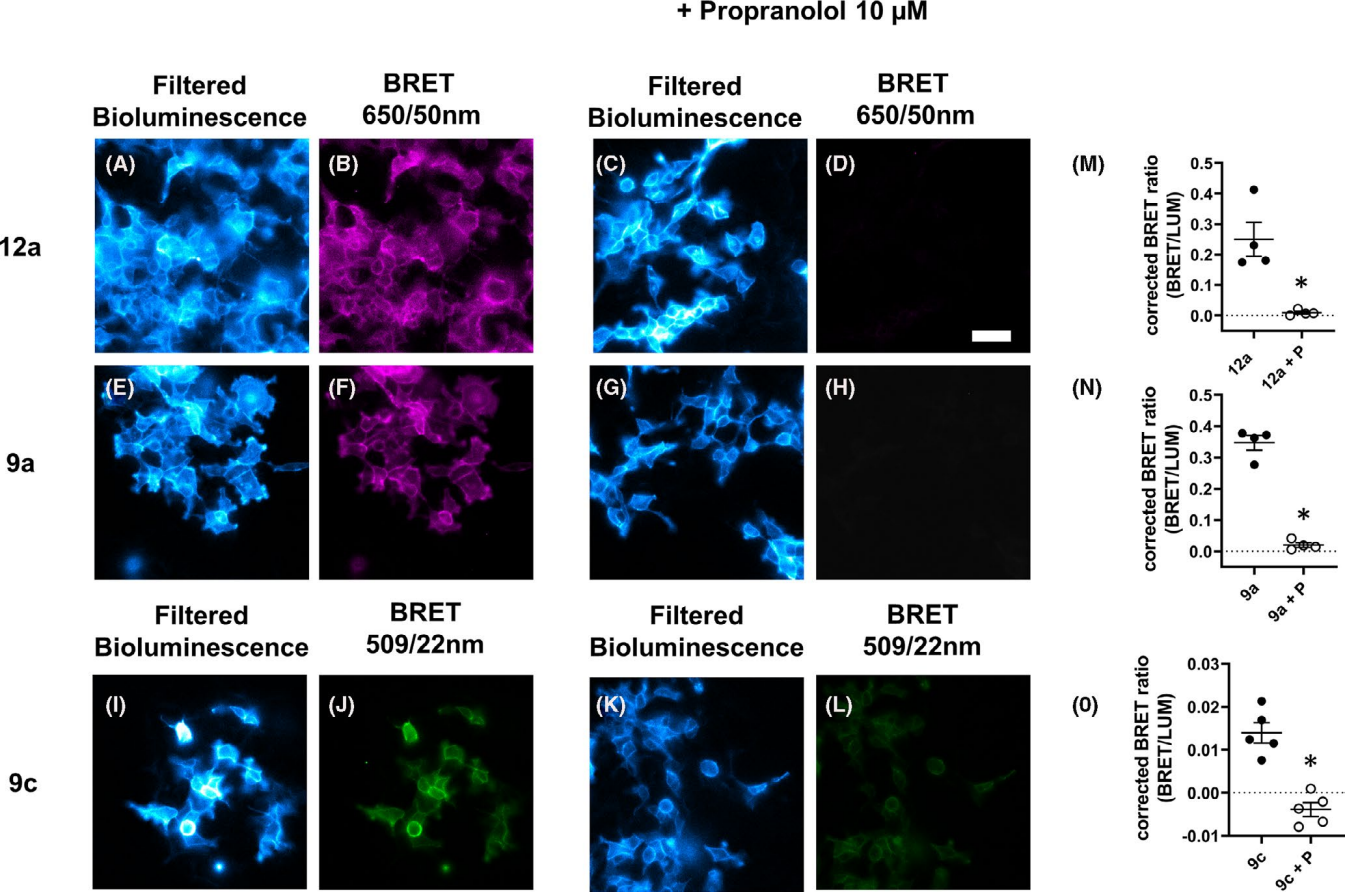
NanoBRET imaging of fluorescent ICI 118,551 derivatives. Cells were incubated with fluorescent ligand (100 nM) 12a (A‐D), 9a (E‐H), and 9c (I‐L) in the absence or presence of 10 µM propranolol before addition of furimazine (5 µM) and imaging. Filtered bioluminescence was captured using a 438/24 band‐pass filter. BRET was captured using a 650/50 nm band‐pass filter or a 509/22 nm band‐pass filter. Mean corrected BRET ratios in the absence (filled circle) or presence (open circle) of propranolol (P; 10 µM) from four separate experiments are illustrated for 12a (M) and 9a (N) and from five separate experiments for 9c (O). Corrected BRET ratios were significantly reduced in the presence of propranolol; paired t‐test *P < .05. Grouped average ±S.E.M. is displayed. Scale bar represents 50 μm

To evaluate whether the proximity requirements (<10 nm) of NanoBRET allowed good localization of fluorescent ICI 118,551 analog binding to both cell surface NLuc‐β_2_ARs and intracellular NLuc‐β_2_ARs in transfected HEK293 T cells, we also undertook bioluminescence imaging using an Olympus LV200 microscope (Figure 5). Filtered luminescence from NLuc‐β_2_ARs was captured using a 428/24 band‐pass filter to allow blue light largely emitted from the N‐terminal nanoluciferase to be monitored. BRET‐emitted light from the red BODIPY‐X‐630/650 fluorophore attached to **9a and 12a** was captured using 650/50 nm band‐pass filter and the BRET‐emitted light from the green BODIPY‐X‐FL fluorophore attached to **9c** was collected using a 509/22 nm band‐pass filter (Figure 5). All three fluorescent ICI 118,551 ligands showed clear NanoBRET binding to the NLuc‐β_2_ARs that was clearly displaceable by 10 μM propranolol (Figure 5). The extent of ligand binding in the presence of 10 μM propranolol appeared higher for the green ligand (**9c**; Figure 5l), but this is partly due to the bleed‐through of nanoluciferase luminescence into the green channel.

### 3.5 Luminescence imaging NLuc‐β_2_ARs in CRISPR/Cas9 genome‐edited HEK293 T cells under endogenous promotion

**FIGURE 6:**
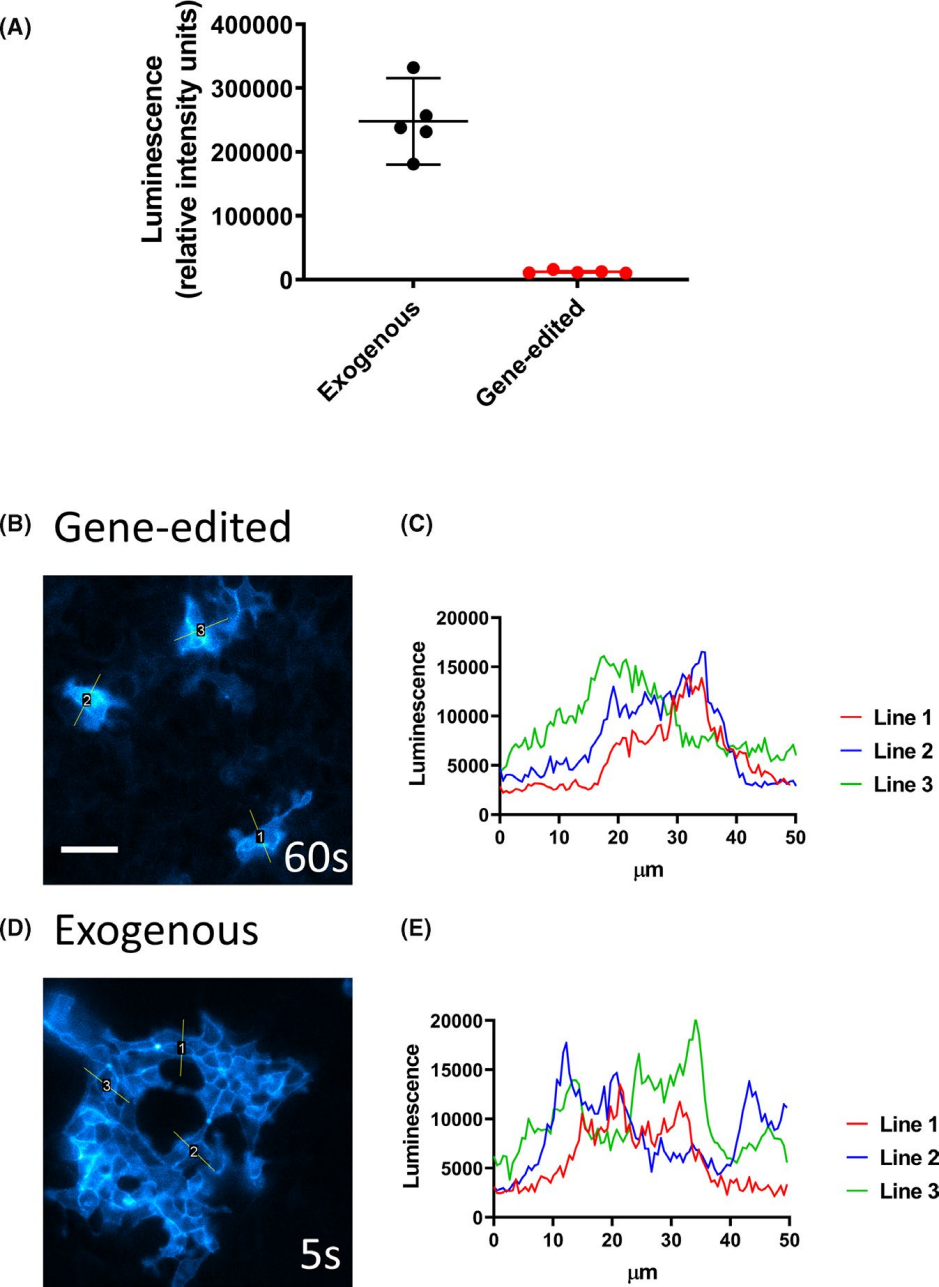
Bioluminescence imaging of HEK293 T cells expressing NLuc‐β_2_AR under endogenous promotion. (A) Relative expression of NLuc‐β_2_AR in the genome‐edited HEK293 T cell line compared to the exogenous HEK293 T cell line. Values are the means from five separate experiments. The lines show the overall mean and 95% confidence limits. (B‐E) Cells expressing the NLuc‐β_2_AR under (​B) endogenous or (D) exogenous promotion were imaged on the Olympus LuminoView 200 (gain of 200) for 60 s or 5 s, respectively, and filtered bioluminescence emission (438/24 band‐pass filter) collected. All other settings were kept constant. Comparative luminescence is illustrated by line profiles (50 µm) for (C) endogenous and (E) exogenous captures. Scale bar represents 50 µm

In HEK293 T cells, the endogenous expression of β_2_‐adrenoceptors is extremely low, and we have previously shown that β_2_ARs tagged with an N‐terminal SNAP label under endogenous promotion are not detectable by standard confocal microscopy in CRISPR/Cas9 genome‐edited HEK293 T cells (Figure S4).^35^ To confirm this low level of expression, we used CRISPR/Cas9 genome editing to incorporate an N‐terminal NLuc tag to the β_2_AR under endogenous promotion as we have described previously.^32^ Quantification of NLuc‐β_2_AR expression, as measured on the PHERAstar FS plate reader, showed a 20.1‐fold higher expression of β_2_ARs in the exogenous stable cell line compared to the genome‐edited HEK293 T cells (Figure 6A). Interestingly, the NLuc‐β_2_AR was clearly detectable in the genome‐edited cells using bioluminescence imaging microscopy (Figure 6B). The signal to noise ratio is much greater for bioluminescence imaging compared to standard confocal imaging and as such imaging at endogenous levels is possible. It must be noted, however, that a longer exposure time (1 min) was required to image the CRISPR/Cas9 genome‐edited NLuc‐β2AR than the transfected cell lines (5 sec, Figure 6B‐E).

### 3.6 NanoBRET binding of 9c and 12a to CRISPR/Cas9 genome‐edited NLuc‐β_2_ARs under endogenous promotion in HEK293 T cells

**FIGURE 7:**
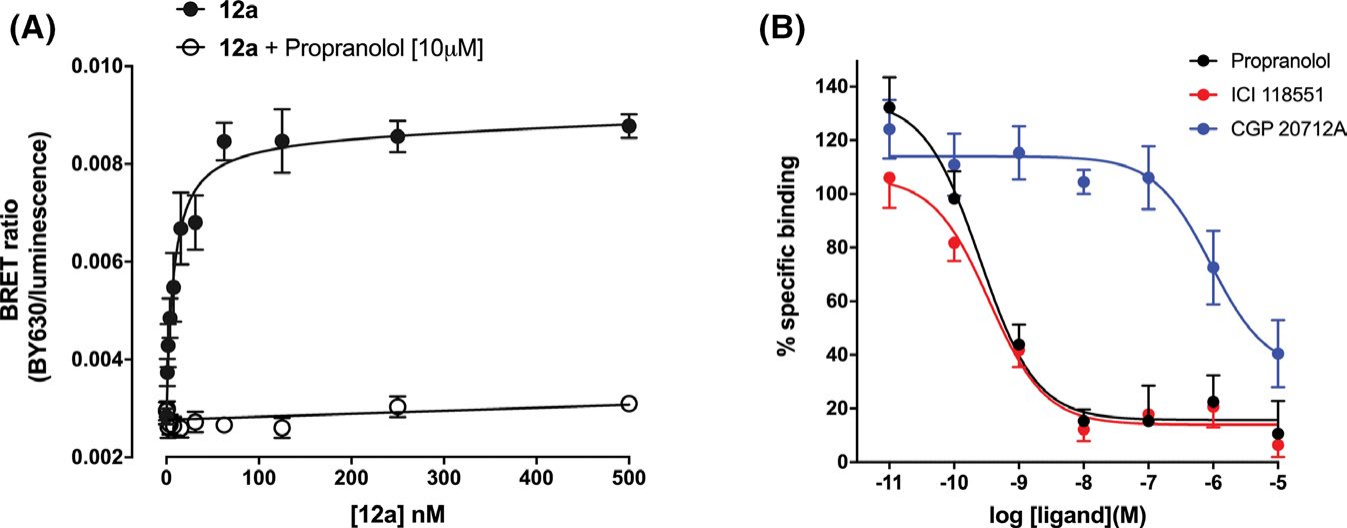
NanoBRET ligand binding for 12a in HEK293 T cells expressing NLuc‐β_2_AR under endogenous promotion. (A) Saturation binding of 12a obtained in the absence (filled circles) and presence (open circles) of 10 µM propranolol. Data are mean ±S.E.M obtained in five separate experiments. (B) Competition of 10 nM 12a in the presence of increasing concentrations of propranolol, ICI 118,551 and CGP 201712A. Data are mean ±S.E.M. from five independent experiments

**FIGURE 8:**
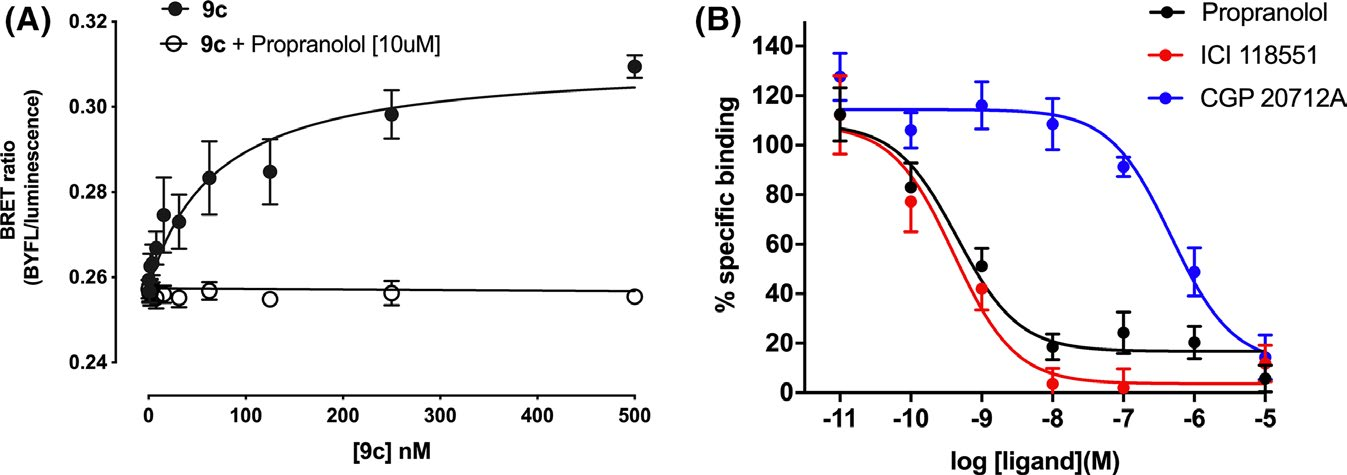
NanoBRET ligand binding for 9c on HEK293 T cells expressing NLuc‐β_2_AR under endogenous promotion. (A) Saturation binding of 9c was obtained in the absence (filled circles) and presence (open circles) of 10 µM propranolol. Data are mean ±S.E.M obtained in six separate experiments. (B) Competition of 10 nM 9c in the presence of increasing concentrations of propranolol, ICI 118,551, and CGP 201712A. Data are mean ±S.E.M. from four (CGP 20712A) or seven (propranolol, ICI 118,551) independent experiments

The huge dynamic range (brightness) of the nanoluciferase luminescence allowed NanoBRET ligand binding of both **9c** and **12a** to be monitored in genome‐edited HEK293 T cells. The PEG‐BODIPY‐X‐630/650 analog of ICI 118,551 (**12a**) showed clear saturable binding with a pK_D_ value of 7.84 ± 0.17 (*n* = 7; Figure 7A), which was similar to the 7.56 ± 0.08 (*n* = 5) determined for **12a** in the transgenic cell line (Table 1). The level of non‐specific binding (obtained in the presence of 10 μM propranolol) was, however, extremely low (Figure 7A). Competition binding experiments in this cell line with **12a** showed the expected β_2_‐selectivity with propranolol (pKi 9.78 ± 0.21; *n* = 5) and salmeterol (8.76 ± 0.21; *n* = 5), and ICI 118,551 (9.70 ± 0.22; *n* = 5) showing high affinity and the β_1_‐selective antagonist CGP 20712A a very low affinity (Figure 7B). Similarly, the βAla‐βAla‐BODIPY‐FL‐X analog of ICI 118,551 (**9c**) also showed clear saturable binding to NLuc‐β_2_ARs in genome‐edited HEK293 T cells (Figure 8A) with a pK_D_ 7.71 ± 0.14 (*n* = 6) that was similar to that measured in transgenic HEK293 T cells (7.48 ± 0.08; Table 1), although the level of non‐specific binding appeared higher than observed with the red ligand (Figure 8A). This is likely to be due to bleed‐through of nanoluciferase luminescence into the green channel (509/22 nm). Competition binding experiments in this cell line with **9c** also showed the expected β_2_‐selectivity with propranolol (pKi 9.55 ± 0.22; *n* = 7) and salmeterol (pKi 8.98 ± 0.27; *n* = 6), and ICI 118,551 (9.69 ± 0.22; *n* = 7) showing high affinity and the β_1_‐selective antagonist CGP 20712A a much lower affinity (6.46 ± 0.12; *n* = 4; Figure 8B).

**FIGURE 9:**
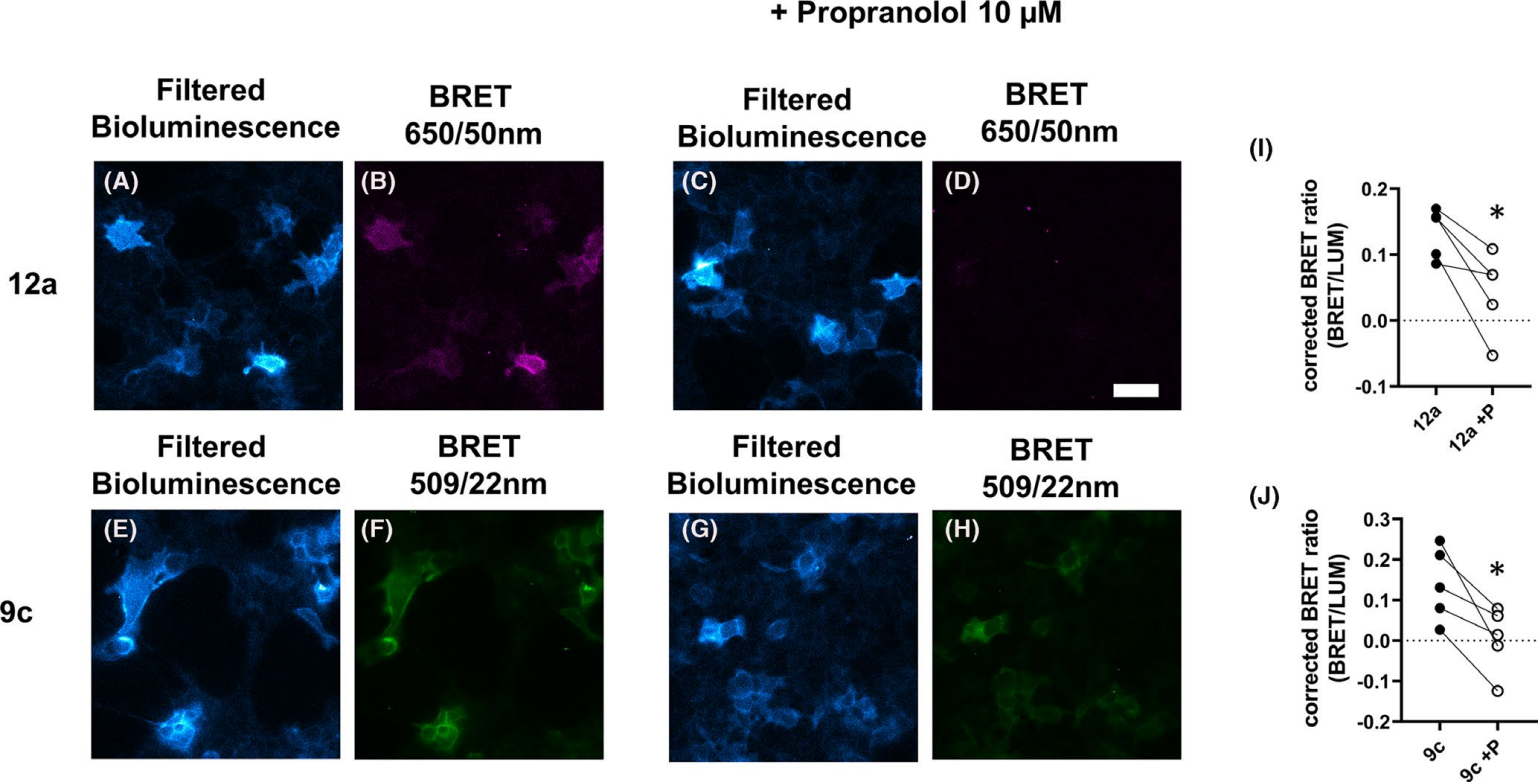
NanoBRET imaging of β_2_AR at endogenous levels. HEK293 T cells expressing NLuc‐β_2_AR under endogenous promotion were incubated with fluorescent ligand (100 nM) 12a (A‐D) or 9c (E‐H) in the absence or presence of 10 µM propranolol before addition of furimazine (5 µM) and imaging. Filtered bioluminescence was captured using a 438/24 band‐pass filter, BRET was captured using a 650/50 nm band‐pass filter or a 509/22 nm band‐pass filter. Mean corrected BRET ratios in the absence or presence of propranolol (P; 10 µM) from five separate experiments are illustrated for 12a (I) and 9c (J). Corrected BRET ratios were significantly reduced in the presence of propranolol; paired t‐test *P < .05. Paired experiments are connected by line. Scale bar represents 50 µm

Finally, we investigated whether NanoBRET ligand binding could be visualized in CRISPR/Cas9 genome‐edited cells using luminescence microscopy. Clear specific binding was detectable with both **12a** and **9c** in these cells (Figure 9), although, as might be expected, the exposure time for both ligands needed to be extended to allow detection. Nevertheless, significant displacement of specific binding by propranolol was detected in paired experiments (Figure 9).

## 4 DISCUSSION

Until recently, the use of NanoBRET to investigate ligand‐binding properties required the exogenous expression of the nanoluciferase‐tagged GPCR of interest, and this can affect how receptors interact and signal. For example, the CXCR4 chemokine receptor has recently been shown to transiently form homodimers at high receptor densities.^46^ β_2_ARs are also known to have a propensity to form dimers and higher order oligomers.^47^ To maintain the normal cellular context, we have recently used CRISPR/Cas9‐mediated homology‐directed repair to insert both luminescent and fluorescent tags onto the N‐terminus of GPCRs into the endogenous genome, where their expression is regulated by endogenous promoters.^30^, ^31^, ^32^, ^35^ In the present manuscript, we have investigated the utility of highly selective fluorescent ligands based on the β_2_AR selective antagonist ICI 118,551^33^ in combination with CRISPR/Cas9 genome editing to investigate ligand binding to endogenous β_2_ARs under the regulation of their native promoters in HEK293 T cells. HEK293 T cells express endogenous β_2_ARs at extremely low levels, which are not detectable by standard confocal microscopy in CRISPR/Cas9 genome‐edited HEK293 T cells expressing SNAP‐tag fluorescent labels [35; Figure S4].

The highly β_2_AR‐selective antagonist ICI 118,551 was used as the starting point for the development of selective β_2_AR fluorescent ligands. We generated a small focused library of nine ligands based on combinations of different linkers and fluorophores. With the exception of ICI 118,551‐Gly‐Ala‐BODIPY‐FL, the remaining fluorescent ICI 118,551 analogs had good affinity (pK_D_ >7) for the β_2_AR with good selectivity over the β_1_AR (Table 1) with the most potent and selective ligands being **8c** (ICI 118,551‐Gly‐Ala‐BODIPY‐FL‐X), **9c** (ICI 118,551 βla‐βAla‐BODIPY‐FL‐X), **12a** (ICI 118,551‐PEG‐BODIPY‐X‐630/650), and **12b** (ICI 118,551‐PEG‐BODIPY‐FL).

**9a** (ICI 118,551‐βAla‐βAla‐BODIPY‐X‐630/650) had the highest affinity at recombinant β_2_ARs (pK_D_ 7.57) but also exhibited significant binding affinity for the β_1_AR (pK_D_ 6.69). Nevertheless, among the red fluorescent ligands, **9a** had the best imaging characteristics (in terms of signal to noise and the extent of non‐specific binding) in recombinant HEK293 T cells and labeling was mostly confined to the cell surface (Figure 4). In contrast, **12a** showed the highest propensity to label intracellular β_2_ARs in HEK293 T cell expressing exogenous receptor. This suggests that a combination of the PEG linker and the BODIPY‐X‐630/650 makes this ICI 118,551 derivative particularly susceptible to crossing the cell membrane to access the intracellular regions of the cell (to bind to intracellular β_2_ARs), or is able to stimulate to some extent receptor endocytosis. Consistent with this latter possibility, ICI 118,551 has been previously reported to stimulate MAP kinase activity via the β_2_ARs in a manner that is dependent on β‐arrestin recruitment to the receptor.^48^ It is also known that β_2_ARs can continue to signal from intracellular endosomes^49^ and so fluorescent ligands, such as **12a,** which appear to be cell permeable and can access endosomal β_2_ARs, may have utility in unraveling the complexities of signaling from intracellular β_2_ARs.

The much lower expression of β_2_ARs in native HEK293 T cells was confirmed when the luminescence detected from NLuc was compared between a stable cell line expressing exogenous NLuc‐β_2_ARs under the control of a human cytomegalovirus (CMV) promoter and CRISPR/Cas9 genome‐edited NLuc‐β_2_ARs under the control of their native promoters (Figure 6). This confirmed our previous observations using this approach with CXCR4 chemokine receptors.^31^ Nevertheless, even at this low expression level in the CRISPR genome‐edited cells, we were able to visualize the expression of NLuc‐β_2_ARs using real‐time luminescence microscopy in a manner which was not possible using conventional confocal microscopy of a similar genome‐editing approach to insert a SNAP‐based fluorescent tag onto the native β_2_ARs.^35^ This shows the huge advantage of using the very bright nanoluciferase and the ability to capitalize on its large dynamic range^50^ to visualize NLuc‐tagged receptors at very low expression levels.

The combination of highly β_2_AR‐selective fluorescent ligands and genome‐edited cells expressing NLuc‐β_2_ARs under the control of their native promoters has enabled real‐time NanoBRET ligand binding to be performed on these receptors for the first time. The high β_2_AR‐selectivity removes any complications caused by the potential of the fluorescent ligand binding to endogenous β_1_ARs and mediating a BRET signal via bystander resonance energy transfer. Furthermore, the ligand‐binding properties in terms of competition by selective β_2_‐ and β_1_‐ ligands in CRISPR/Cas9‐edited cells were very similar to those obtained for the transgenic cell line. The pK_D_ values obtained for both **12a** (ICI 118,551‐PEG‐BODIPY‐X‐630/650) and the green ligand **9c** (ICI 118,551 βAla‐βAla‐BODIPY‐FL‐X) were similar to the values obtained with the same fluorescent ligand in the transfected cell line.

## 5 CONCLUSION

In summary, the present manuscript has generated some high β_2_AR‐selective fluorescent ligands based on ICI 118​,551 that have been used to investigate ligand binding to β_2_ARs in CRISPR/Cas9 genome‐edited HEK293 T cells where the endogenous receptor expression is extremely low. We have used these ligands to undertake for the first time real‐time ligand binding to the native β_2_ARs to provide both quantitative data on ligand‐binding characteristics and to allow visualization of the ligand‐binding interactions using NanoBRET luminescence imaging. The fluorescent ligands studied have different abilities to label intracellular β_2_ARs and these should be valuable tools to help unravel the complexities of β_2_AR pharmacology in endosomal compartments where these receptors can continue to signal after agonist‐induced receptor endocytosis.

## 6 ETHICS APPROVAL STATEMENT

No ethical approvals were required for this work.

## DISCLOSURE

The authors declare no conflict of interest.

## AUTHOR CONTRIBUTIONS

Participated in research design: *JG*, *SJM*, *MS*, *SJB*, *CWW*, *BK*, *SJH*. Conducted experiments: *JG*, *SJM*, *MS*, *CWW*. Performed data analysis: *JG*, *MS*, *CWW*. Wrote or contributed to the writing of the manuscript: *JG*, *SJM*, *MS*, *JW*, *SJB*, *CWW*, *BK*, *SJH*.

## Supporting information

Supplementary Material

## Data Availability

The data that support the findings of this study are available from the corresponding author upon reasonable request.
