# Supplementary material for: Subtype selective fluorescent ligands based on ICI 118,551 to study the human β2‐adrenoceptor in CRISPR/Cas9 genome‐edited HEK293T cells at low expression levels
Source: Pharmacol Res Perspect. 2021 May 18;9(3):e00779. doi: 10.1002/prp2.779 (PMC8130569; doi:10.1002/prp2.779)
Supplement: Supplementary file 1 — Supplementary Material [file PRP2-9-e00779-s001.pdf]

Supplementary Information.

Sub-type selective fluorescent ligands based on ICI 118,551 to study the human β2-adrenoceptor in CRISPR/Cas9 genome-edited HEK293T cells.

Joëlle Goulding [1,2,†], Sarah J. Mistry [2,3,†], Mark Soave [1,2], Jeanette Woolard [1,2], Stephen J. Briddon [1,2], Carl W White[1,2,4,5], Barrie Kellam [2,3,*], Stephen J. Hill [1,2,*]

[†] JG and SJM contributed equally to this work.

[1]Division of Physiology, Pharmacology and Neuroscience, School of Life Sciences, University of Nottingham, Nottingham, NG7 2UH, UK.

[2]Centre of Membrane Proteins and Receptors (COMPARE), University of Birmingham and University of Nottingham, Midlands, UK.

[3]School of Pharmacy, University of Nottingham, Nottingham, NG7 2UH, UK.

[4]Harry Perkins Institute of Medical Research and Centre for Medical Research, The University of Western Australia, QEII Medical Centre, Nedlands, Western Australia 6009, Australia.

[5]Australian Research Council Centre for Personalised Therapeutics Technologies, Australia

*Correspondence to Professor S J Hill; Stephen.hill@nottingham.ac.uk or Professor B Kellam; barrie.kellam@nottingham.ac.uk

Chemistry

Abbreviations; Boc_2O, di-*tert*-butyl dicarbonate ; BODIPY 630/650-X-SE, 6-(((4,4-difluoro-5-(2-thienyl)-4-bora-3a,4a-diaza-s-indacene-3-yl)-styryloxy)acetyl)aminohexanoic acid succinimidyl ester; BODIPY-FL-X-SE, 6-((4,4-difluoro-5,7-dimethyl-4-bora-3a,4a-diaza-s-indacene-3-propionyl)amino)hexanoic acid, succinimidyl ester; BODIPY-FL-SE, [1-[3-[5-[(3,5-dimethyl-2*H*-pyrrol-2-ylidene-κ-*N*)methyl]-1*H*-pyrrol-2-yl-κ-*N*]-1-oxopropoxy]-2,5-pyrrolidinedionato]difluoro-,(T-4)-Boron; DCM, dichloromethane; DIAD, diisoproyl azodicarboxylate; DIPEA, diisopropylethylamine; DMF, *N,N*-dimethylformamide; HBTU; *N,N,N′,N′*-Tetramethyl-*O*-(1*H*-benzotriazol-1-yl)uronium hexafluorophosphate; *m*-CPBA, ***meta*-chloroperbenzoic acid**; TFA, trifluoroacetic acid.

Chemistry synthesis.

The synthesis of congeners (**8a** to **12c**, **Supplementary Figure 1**) proceeded as follows; firstly, commercially available 7-methyl-4-indanol (**1**) underwent a Mitsunobu reaction with *E*-crotyl alcohol to afford alkene **2**. This was oxidised with *m*-CPBA to afford the corresponding oxirane **3**, which underwent ring-opening with either an *N*-Boc-protected poly(ethylene glycol) (PEG) linker (obtained *via* mono *N*-Boc-protection of commercially available 2,2′-(ethylenedioxy)diethylamine with Boc_2O as previously described (Friscourt et al., 2012)) or *N*-Boc-protected ethylene diamine, to afford *N*-Boc protected compounds **4** and **10** respectively. *N*-Boc-deprotection of **4** and **10** under acidic conditions afforded either hydrochloride (**5**) or 2,2,2-trifluoroacetate (**11**) salts of the corresponding primary amines.

Compound **5** subsequently underwent two cycles of *N*-Boc-amino acid coupling (in the presence of *N,N,N′,N′*-tetramethyl-*O*-(1*H*-benzotriazol-1-yl)uronium hexafluorophosphate (HBTU) and Hunig's base)/acidic *N*-Boc-deprotection to give **7a** and **7b**. Finally, these amine salts were acylated with commercially available 6-(((4,4-difluoro-5-(2-thienyl)-4-bora-3a,4adiaza-s-indacene-3-yl)styryloxy)acetyl)amino hexanoic acid succinimidyl ester (BODIPY-630/650-X-SE), 6-((4,4-difluoro-5,7-dimethyl-4-bora-3a,4a-diaza-s-indacene-3-propionyl)amino)hexanoic acid, succinimidyl ester (BODIPY-FL-X-SE) or 4,4-difluoro-5,7-dimethyl-4-bora-3a,4a-diaza-s-indacene-3-propionic acid, succinimidyl ester (BODIPY-FLSE)

to afford fluorescent ligands **8a** to **12c** in excellent yields**.** The crude fluorescent ligands were purified by preparative thin layer chromatography (pTLC) or semi-preparative reverse phase high performance liquid chromatography (RP-HPLC). Compound identity and purity of ≥95% was confirmed by HRMS spectrometry (ES-TOF) and RP-HPLC (with photodiode array detection between 190 and 800nm) respectively.

***tert*-Butyl {2-[2-(2-aminoethoxy)ethoxy]ethyl}carbamate.**

To 2-2'-(ethylenedioxy)-*bis*-ethylamine (3.00g, 20.2 mmol, 3.0 eq) and DIPEA (1.18 mL, 6.75 mmol, 1.0 eq) in DCM (45.0 mL) at 0°C under N_2, a solution of Boc_2O (1.473 g, 6.75 mmol, 1.0 eq) in DCM (30.0 mL) was added via a dropping funnel. After stirring for 22 hours at room temperature the solvent was removed *in vacuo*. The resulting residue was purified by column chromatography (9:1 DCM/MeOH) to afford the title compound as a pale yellow oil 1.36 g, 81%. ^{1}H NMR (400 MHz, $CDCl_3$) δ = 5.18 (s, 1H), 3.61 (s, 4H), 3.56-3.48 (m, 4H), 3.36-3.25 (m, 2H), 2.87 (t, *J* = 5.4Hz, 2H), 1.83 (broad s, 2H), 1.43 (s, 9H). ^{13}C NMR (101 MHz, $CDCl_3$) δ = 156.2, 79.3, 73.4, 70.4, 70.3, 50.8, 41.8, 40.5, 28.6. HRMS (ESI-TOF) *m/z* calc. for $C_{11}H_{25}N_2O_4$ $[M+H]^+$; 249.1809, found 249.1813.

1-(7-Methylindan-4-yloxy) but-*trans*-2-ene) (2).

7-Methyl-4-indanol (3.00 g, 2.0 mmol 1.0 eq), *trans*-crotyl alcohol (2.19 g, 3.0 mmol, 1.5 eq) and triphenylphosphine (7.96 g, 3.0 mmol, 1.5 eq) were dissolved in THF (100.0 mL) and cooled to 0°C. DIAD (6.14 g, 3.0 mmol, 1.5 eq) was added dropwise and the mixture was stirred at room temperature for 6 days (further DIAD (1.0 eq) was added after 24 and 48 hours). The solvent was removed *in vacuo* and pet. ether was added to precipitate out the triphenylphosphine oxide. The triphenylphosphine was removed by vacuum filtration and after concentration of the filtrate, the crude product was purified by column chromatography on silica (0.1:9.9 to 1:9 Et_2O/pet. ether). The title compound was afforded as a clear colourless oil (2.50 g, 61%) ^{1}H NMR (400 MHz, $CDCl_3$) δ = 6.91 (d, *J* = 8.0 Hz, 1H), 6.60 (d, *J* = 8.1 Hz, 1H), 5.90 – 5.79 (m, 1H), 5.78 – 5.68 (m, 1H), 4.46 (dt, *J* = 5.6 Hz, 1.3 Hz, 2H), 2.91 (t, *J* = 7.5 Hz, 2H), 2.84 (t, *J* = 7.5 Hz, 2H), 2.21 (s, 3H), 2.08 (quintet, *J* = 7.5 Hz, 2H), 1.76 (dq, *J* = 6.4

Hz, 1.3 Hz). ^{13}C NMR (101 MHz, $CDCl_3$) δ = 153.5, 145.0, 131.9, 129.6, 127.8, 126.9, 126.1, 109.8, 77.5, 77.4, 77.2, 76.8, 68.9, 32.1, 30.0, 24.7, 18.5, 18.0.

2-Methyl-3-(7-methylindan-4-oxymethyl)oxirane (3).

To compound 1-(7-methylindan-4-yloxy) but-*trans*-2-ene) (**2**) (1.00 g, 4.9 mmol, 1.0 eq) in DCM (16.0 mL) at 0°C *m*-CPBA (1.14 g, 6.6 mmol, 1.3 eq) was added portion-wise. The mixture was warmed to room temperature and was stirred for 5 days (a further 0.5 eq and 0.2 eq *m*-CPBA added after 1 day and 4 days respectively). 5% sodium thiosulfate$_{(aq)}$ was added and stirred for 20 minutes. The organic layer was washed twice with 5% sodium thiosulfate$_{(aq)}$ and then brine and dried over $MgSO_4$. The solvent was removed *in vacuo* and the crude product was purified by column chromatography on silica (0.5:9.5 EtOAc/pet. ether) The title product was afforded an off-white solid (0.441 g, 41%). ^1H NMR (400 MHz, $CDCl_3$) δ = 6.90 (d, *J* = 8.2 Hz, 1H), 6.58 (d, *J* = 8.2 Hz, 1H), 4.16 (dd, *J* = 11.1, 3.4 Hz, 1H), 3.99 (dd, *J* = 11.2, 5.1 Hz, 1H), 3.11 – 3.01 (m, 2H), 2.91 (t, *J* = 7.5 Hz, 2H), 2.83 (t, *J* = 7.5 Hz, 2H), 2.20 (s, 3H), 2.08 (quintet, *J* = 7.5 Hz, 2H), 1.38 (d, *J* = 5.1 Hz, 3H). ^{13}C NMR (101 MHz, $CDCl_3$) δ = 153.3, 145.2, 132.0, 127.9, 126.7, 109.7, 68.6, 57.5, 52.7, 32.1, 29.9, 24.7, 18.6, 17.5. HRMS (ESI-TOF) *m/z* calc. for $C_{14}H_{19}O_2$ $[M+H]^+$; 219.1380, found 219.1378 and 241.1199 [M+Na].

(±)-*tert*-Butyl-(2-((3-hydroxy-4-((7-methyl-2,3-dihydro-1*H*-inden-4-yl)oxy)butan-2-yl)amino)ethyl)carbamate (4).

To a solution of 2-methyl-3-(7-methylindan-4-oxymethyl)oxirane (**3**) (0.30 g, 1.4 mmol, 1.0 eq) in MeOH (5.0 mL), *N*-Boc-ethylene diamine (1.10 g, 6.9 mmol, 5.0 eq) was added and the mixture was heated at reflux for 2 days. The solvent was removed *in vacuo* and the crude product was purified by column chromatography (9.5:0.4:0.1 DCM/MeOH/1N NH_3 in MeOH) to afford a yellow oil which slowly crystallised (0.35 g, 67%). ^1H NMR (400 MHz, $CDCl_3$) δ = 6.90 (d, *J* = 8.1 Hz, 1H), 6.59 (d, *J* = 8.1 Hz, 1H), 4.98 (t, *J* = 8.0 Hz, 1H) 4.19 – 3.91 (m, 3H), 3.40 – 3.19 (m, 2H), 3.09 – 2.78 (m, 6H), 2.71 (dt, *J* = 11.9, 5.9 Hz, 1H), 2.19 (s, 3H), 2.07 (quintet, *J* = 7.6 Hz, 2H), 1.44 (s, 9H), 1.14 (d, *J* = 6.6 Hz, 3H). ^{13}C NMR (101 MHz, $CDCl_3$) δ = 156.4, 153.3, 145.1, 131.6, 128.0, 126.6, 109.5, 79.6, 70.7, 69.5, 55.4, 53.6, 47.1, 41.0,

40.7, 32.1, 30.0, 28.6, 24.6, 18.5, 15.1. LCMS *m/z* calc. for $C_{21}H_{35}N_2O_4$ $[MH]^+$ 379.3; found; 379.1, t_R = 2.33 min.

(±)-3-((2-Aminoethyl)amino)-1-((7-methyl-2,3-dihydro-1*H*-inden-4-yl)oxy)butan-2-ol dihydrochloride (5).

To compound (±)-*tert*-butyl-(2-((3-hydroxy-4-((7-methyl-2,3-dihydro-1*H*-inden-4-yl)oxy)butan-2-yl)amino)ethyl)carbamate (**4**) (0.34 g, 0.90 mmol, 1.0 eq) in Et_2O (2.0 mL) 4M HCl in dioxane (2.0 mL) was added. A thick white precipitate formed and after stirring for 24 hours it was collected by vacuum filtration (0.28 g, 88%). ^{1}H NMR (400 MHz, MeOD) δ = 6.91 (d, *J* = 8.1 Hz, 1H), 6.68 (d, *J* = 8.1 Hz, 1H), 4.43 (td, *J* = 6.2, 2.7 Hz, 1H), 4.10 (dd, *J* = 9.8, 6.1 Hz, 1H), 3.98 (dd, *J* = 9.9, 6.5 Hz, 1H), 3.74 – 3.50 (m, 1H), 3.57 – 3.38 (m, 4H), 2.90 (t, *J* = 7.5 Hz, 2H), 2.84 (t, *J* = 7.5 Hz, 2H), 2.19 (s, 3H), 2.08 (quintet, *J* = 7.5 Hz, 2H), 1.42 (d, *J* = 6.7 Hz, 3H). ^{13}C NMR (101 MHz, MeOD) δ = 154.2, 145.8, 132.4, 129.1, 127.7, 110.7, 69.6, 68.3, 58.2, 43.2, 36.9, 32.7, 30.7, 25.6, 18.4, 10.5. LCMS *m/z* calc. for $C_{16}H_{27}N_2O_2$ $[MH]^+$; 279.2 found 279.1, t_R = 1.87 min.

General method A; synthesis of analogues 2-amino-*N*-(2-(((2*S*)-3-hydroxy-4-((7-methyl-2,3-dihydro-1*H*-inden-4-yl)oxy)butan-2-yl)amino)ethyl)acetamide ditrifluoroacetate (6a) to (±)-(3-((3-((2-((3-hydroxy-4-((7-methyl-2,3-dihydro-1*H*-inden-4-yl)oxy)butan-2-yl)amino)ethyl)amino)-3-oxopropyl)amino)-3-oxopropyl)amine ditrifluoroacetate (7b) via HBTU coupling and Boc deprotection cycles with (6a) as an example

2-Amino-*N*-(2-(((2*S*)-3-hydroxy-4-((7-methyl-2,3-dihydro-1*H*-inden-4-yl)oxy)butan-2-yl)amino)ethyl)acetamide ditrifluoroacetate (6a).

To Boc-Gly-OH (0.09 g, 0.52 mmol, 1.0 eq) in DMF (2.0 mL) HBTU (0.24 g, 0.63 mmol, 1.2 eq) and DIPEA (0.46 mL, 2.62 mmol, 5.0 eq) were added. After stirring for 15 minutes, (±)-3-((2-aminoethyl)amino)-1-((7-methyl-2,3-dihydro-1*H*-inden-4-yl)oxy)butan-2-ol dihydrochloride (**5**) (0.18 g, 0.52 mmol, 1.0 eq) was added. The mixture was stirred at room temperature for 26 hours and then partitioned between a 1:1 mixture of sat. $NaHCO_3$/ water and EtOAc. The organic layer was washed again with sat. $NaHCO_3$/ water and then twice with brine. The solvent was removed *in vacuo* to afford a colourless residue which was purified by column

chromatography (9:1 DCM/1N NH_3 in MeOH). The Boc-protected product was afforded as an off-white solid (0.15 g, 64%). ^1H NMR (400 MHz, MeOD) δ = 6.87 (d, *J* = 8.2 Hz, 1H), 6.62 (d, *J* = 8.1 Hz, 1H), 4.03 – 3.93 (m, 3H), 3.64 (s, 2H), 3.37 – 3.32 (m, 5H), 2.99 – 2.71 (m, 7H), 2.17 (s, 3H), 2.06 (quintet, *J* = 7.5 Hz, 2H), 1.44 (s, 9H), 1.13 (d, *J* = 6.6 Hz, 3H). ^{13}C NMR (101 MHz, MeOD) δ = 172.9, 158.4, 154.7, 145.6, 132.4, 129.0, 127.1, 110.4, 80.8, 72.1, 70.8, 56.0, 49.9, 47.1, 44.7, 39.9, 32.8, 30.7, 28.7, 25.6, 18.5, 14.6. LCMS *m/z* calc. for $C_{23}H_{38}N_3O_5$ $[MH]^+$; 436.3 found; 436.3, t_R = 2.30 min. The white solid was then deprotected with TFA (0.5mL)/ DCM (0.5 mL) to afford the title compound as a pale yellow oil, which after concentration *in vacuo*, was used directly in the next step. LCMS *m/z* calc. for $C_{18}H_{30}N_3O_3$ $[MH]^+$; 336.2 found; 336.1, t_R = 1.22 min

(±)-(3-((2-((3-Hydroxy-4-((7-methyl-2,3-dihydro-1*H*-inden-4-yl)oxy)butan-2-yl)amino)ethyl)amino)-3-oxopropyl)amine dihydrochloride (6b). The title compound was prepared according to the procedure described in general method A using reagents in the following quantities: Boc-β-Ala-OH (50.0 mg, 0.26 mmol, 1.0 eq), (±)-3-((2-aminoethyl)amino)-1-((7-methyl-2,3-dihydro-1*H*-inden-4-yl)oxy)butan-2-ol dihydrochloride (**5**) (92.0 mg, 0.26 mmol, 1.0 eq), HBTU (0.119 g, 0.31 mmol, 1.2 eq), DIPEA (0.23 mL, 1.31 mmol, 5.0 eq) and DMF (2.0 mL). After the addition of a 1:1 mixture of sat. $NaHCO_3$/ water a white precipitate formed. This was collected by vacuum filtration, washed with water and dried. (0.06 g, 53%). ^1H NMR (400 MHz, MeOD) δ = 6.89 (d, *J* = 8.1 Hz, 1H), 6.64 (d, *J* = 8.1 Hz, 1H), 4.05 – 3.93 (m, 3H), 3.31 – 3.26 (m, 2H), 2.99 – 2.70 (m, 7H), 2.32 (t, *J* = 6.8 Hz, 2H), 2.19 (s, 3H), 2.07 (quintet, *J* = 7.5 Hz, 2H), 1.44 (s, 9H), 1.14 (d, *J* = 6.5 Hz, 3H). ^{13}C NMR (101 MHz, MeOD) δ = 174.2, 158.3, 154.7, 145.6, 132.4, 129.0, 127.1, 110.4, 80.1, 72.5, 70.8, 56.0, 47.2, 40.2, 38.0, 37.3, 32.8, 30.7, 28.8, 25.6, 18.5, 15.0. LCMS *m/z* calc. for $C_{24}H_{40}N_3O_5$ $[MH]^+$; 450.3 found; 450.4, t_R = 2.33 min. Boc deprotection with 4M HCl in dioxane (0.5 mL)/diethyl ether (0.5 mL) afforded an off-white sticky solid (quantitative), which was used directly in the next step. LCMS *m/z* calc. for $C_{19}H_{32}N_3O_3$ $[MH]^+$; 350.2 found; 350.1, t_R = 1.88 min

(±)-((2*S*)-1-((2-((2-((3-Hydroxy-4-((7-methyl-2,3-dihydro-1*H*-inden-4-yl)oxy)butan-2-yl)amino)ethyl)amino)-2-oxoethyl)amino)-1-oxopropan-2-yl)amine ditrifluoroacetate (7a).

The title compound was prepared according to the procedure described in general method A using reagents in the following quantities: Boc-Ala-OH (31.8 mg, 0.17 mmol, 1.0 eq), 2-amino-*N*-(2-(((2*S*)-3-hydroxy-4-((7-methyl-2,3-dihydro-1*H*-inden-4-yl)oxy)butan-2-yl)amino)ethyl)acetamide ditrifluoroacetate (**6a**) (94.4 mg, 0.17 mmol, 1.0 eq), HBTU (76.5 mg, 0.20 mmol, 1.2 eq), DIPEA (0.147 mL, 0.84 mmol, 5.0 eq), DMF (2.0 mL). Purification by column chromatography (9:1 DCM/1N NH_3 in MeOH) afforded the Boc-protected compound as a white foam (0.06 g, 65%). ^{1}H NMR (400 MHz, MeOD) δ = 6.87 (d, *J* = 8.2 Hz, 1H), 6.62 (d, *J* = 8.2 Hz, 1H), 4.08 – 3.90 (m, 4H), 3.87 – 3.71 (m, 2H), 3.53 – 3.39 (m 1H), 3.30 – 3.24 (m, 1H), 3.00 – 2.91 (m, 1H), 2.90 – 2.74 (m, 7H), 2.17 (s, 3H), 2.05 (quintet, *J* = 7.5 Hz, 2H), 1.45 (s, 9H), 1.31 (d, *J* = 7.2, 3H), 1.13 (d, *J* = 6.6 Hz, 3H). ^{13}C NMR (101 MHz, MeOD) δ = 176.7, 172.0, 158.3, 154.7, 145.6, 132.4, 129.0, 127.1, 110. 5, 80.9, 72.0, 70.8, 56.0, 52.3, 47.0, 43.7, 40.0, 32.8, 30.7, 28.8, 25.6, 18.5, 17.6, 14.6. LCMS *m/z* calc. for $C_{26}H_{43}N_4O_6$ $[MH]^+$; 507.3 found; 507.3, t_R = 2.30 min. Boc deprotection with TFA (0.5 mL)/DCM (0.5 mL) afforded an orange oil (quantitative) which was used directly in the next step. LCMS *m/z* calc. for $C_{21}H_{35}N_4O_4$ $[MH]^+$; 407.3 found; 407.2, t_R = 1.85 min.

(±)-(3-((3-((2-((3-Hydroxy-4-((7-methyl-2,3-dihydro-1*H*-inden-4-yl)oxy)butan-2-yl)amino)ethyl)amino)-3-oxopropyl)amino)-3-oxopropyl)amine ditrifluoroacetate (7b).

The title compound was prepared according to the procedure described in general method A using reagents in the following quantities: Boc-β-Ala-OH (23.0 mg, 0.12 mmol, 1.0 eq), (±)-(3-((2-((3-hydroxy-4-((7-methyl-2,3-dihydro-1*H*-inden-4-yl)oxy)butan-2-yl)amino)ethyl)amino)-3-oxopropyl)amine dihydrochloride (**6b**) (51.4 mg, 0.12 mmol, 1.0 eq), HBTU (55.3 mg, 0.15 mmol, 1.2 eq), DIPEA (0.106 mL, 0.61 mmol, 5.0 eq), DMF (2.0 mL). Purification by column chromatography (9:1 DCM/1N NH_3 in MeOH) afforded the Boc-protected compound as an off-white solid (34.0 mg, 54%). ^{1}H NMR (400 MHz, MeOD δ = 6.88 (d, *J* = 8.1 Hz, 1H), 6.63 (d, *J* = 8.1 Hz, 1H), 4.15 – 3.92 (m, 3H), 3.45 – 3.34 (m, 3H), 3.30 – 3.23 (m, 3H), 3.16 – 3.07 (m,

1H), 3.01 – 2.78 (m, 6H), 2.34 (q, *J* = 6.7 Hz, 4H), 2.17 (s, 3H), 2.06 (quintet, *J* = 7.5 Hz, 3H), 1.42 (s, 9H), 1.19 (d, *J* = 6.6 Hz, 4H). ^{13}C NMR (101 MHz, MeOD) δ = 154.5, 145.7, 132.4, 129.0, 127.9, 127.3, 110.5, 70.5, 69.8, 56.5, 47.2, 39.5, 38.1, 37.4, 37.1, 37.0, 32.8, 30.7, 28.7, 25.6, 18.5. LCMS *m/z* calc. for calcd for $C_{27}H_{45}N_4O_6$ [MH]$^+$; 521.3 found; 521.2, t_R = 2.30 min. Boc-deprotection with TFA (0.5 mL)/DCM (0.5 mL) afforded an off-white sticky solid (quantitative) which was used directly in the next step LCMS *m/z* calc. for $C_{32}H_{37}N_4O_4$ [MH]$^+$; 421.3 found; 421.0, t_R = 1.87 min.

(±)-3-((2-(2-(2-Aminoethoxy)ethoxy)ethyl)amino)-1-((7-methyl-2,3-dihydro-1*H*-inden-4-yl)oxy)butan-2-ol dihydrochloride (11).

To a solution of 2-methyl-3-(7-methylindan-4-oxymethyl)oxirane (**3**) (0.10 g, 0.46 mmol, 1.0 eq) in MeOH (3.0 mL), *tert*-Butyl {2-[2-(2-aminoethoxy)ethoxy]ethyl}carbamate (0.57 g, 2.3 mmol, 5.0 eq) was added and the mixture was heated at reflux for 3 days. The solvent was removed *in vacuo* and the crude residue was purified by column chromatography (1:9 MeOH/DCM) to afford a yellow oil (0.07 g, 32%). ^{1}H NMR (400 MHz, $CDCl_3$) δ = 6.90 (d, *J* = 7.8 Hz, 1H), 6.60 (d, *J* = 8.1 Hz, 1H), 5.24 (s, 1H) 4.09 – 3.93 (m, 2H), 3.68 – 3.57 (m, 5H), 3.53 (t, *J* = 5.2 Hz, 2H), 3.31 (q, *J* = 5.6 Hz, 2H), 3.06 – 2.93 (m, 2H), 2.87 (t, *J* = 7.5 Hz, 2H), 2.82 (t, *J* = 7.5 Hz, 3H), 2.12 – 2.01 (m, 4H), 1.43 (s, 9H), 1.13 (d, *J* = 6.6 Hz, 3H). ^{13}C NMR (101 MHz, $CDCl_3$) δ = 156.2, 153.3, 145.0, 131.6, 128.0, 126.5, 109.4, 79.3, 70.5, 70.3, 70.3, 70.2, 69.4, 55.5, 46.8, 40.5, 32.1, 29.9, 28.6, 24.6, 18.5, 15.0. LCMS *m/z* calc. for $C_{21}H_{35}N_2O_4$ [MH]$^+$; 467.3, found 467.1, t_R = 2.48 min. Boc-deprotection with 4M HCl in dioxane (0.5mL)/diethyl ether (0.5 mL) afforded a brown oil, which after concentration *in vacuo*, was used directly in the next step. LCMS *m/z* calc. for $C_{20}H_{35}N_2O_4$ [MH]$^+$; 367.3 found; 367.2, t_R = 1.90 min

General method B; Synthesis of analogues (±)-((2*S*)-1-((2-((2-((3-hydroxy-4-((7-methyl-2,3-dihydro-1*H*-inden-4-yl)oxy)butan-2-yl)amino)ethyl)amino)-2-oxoethyl)amino)-1-oxopropan-2-yl)-6-((4-(2-(4,4-difluoro-4,4a-dihydro-5-(thiophen-2-yl)-4-bora-3a,4a-diaza-s-indacene-3-yl)vinyl)phenoxy)acetamido)-hexanamide (8a) to (±)-*N*-(2-(2-(2-((3-hydroxy-4-((7-methyl-2,3-dihydro-1*H*-inden-4-yl)oxy)butan-2-yl)amino)ethoxy)ethoxy)ethyl)-4,4-difluoro-5,7-dimethyl-4-

bora-3a,4a-diaza-s-indacene- 3-propionamide (12c) via fluorophore labelling with the appropriate fluorophore-NHS ester using (8a) as an example

(±)-((2*S*)-1-((2-((2-((3-Hydroxy-4-((7-methyl-2,3-dihydro-1*H*-inden-4-yl)oxy)butan-2-yl)amino)ethyl)amino)-2-oxoethyl)amino)-1-oxopropan-2-yl)-6-((4-(2-(4,4-difluoro-4,4a-dihydro-5-(thiophen-2-yl)-4-bora-3a,4a-diaza-s-indacene-3-yl)vinyl)phenoxy)acetamido)-hexanamide (8a).

To (±)-((2*S*)-1-((2-((2-((3-hydroxy-4-((7-methyl-2,3-dihydro-1*H*-inden-4-yl)oxy)butan-2-yl)amino)ethyl)amino)-2-oxoethyl)amino)-1-oxopropan-2-yl)amine ditrifluoroacetate (**7a**) (1.9 mg, 3.0 µmol, 2.0 eq) in DMF (1.0 mL), DIPEA (1.3 µL, 7.6 µmol, 5.0 eq) and BODIPY-630/650-X-SE (1.0 mg, 1.5 µmol, 1.0 eq) were added. The mixture was stirred at room temperature with the exclusion of light for 11 hours and the DMF was removed. Purification by semi-prep HPLC (method A) afforded a blue solid (1.4 mg, 100%). HRMS (ESI-TOF) *m/z* calcd for $C_{50}H_{61}BF_2N_7O_7S^+$ $[MH]^+$; 952.4409 found; 952.4417. Analytical RP-HPLC; t_R = 19.96 mins, purity = 98%

(±)-((2*S*)-1-((2-((2-((3-Hydroxy-4-((7-methyl-2,3-dihydro-1*H*-inden-4-yl)oxy)butan-2-yl)amino)ethyl)amino)-2-oxoethyl)amino)-1-oxopropan-2-yl)-4,4-difluoro-5,7-dimethyl-4-bora-3a,4a-diaza-s-indacene- 3-propionamide (8b).

The title compound was prepared according to the procedure described in general method B using reagents in the following quantities: (±)-((2*S*)-1-((2-((2-((3-hydroxy-4-((7-methyl-2,3-dihydro-1*H*-inden-4-yl)oxy)butan-2-yl)amino)ethyl)amino)-2-oxoethyl)amino)-1-oxopropan-2-yl)amine ditrifluoroacetate (**7a**) (1.6 mg, 2.57 µmol, 2.0 eq), BODIPY-FL-SE (0.5 mg, 1.28 µmol, 1.0 eq), DIPEA (1.12 µL, 6.42 µmol, 5.0 eq), DMF (1.0 mL). Purification by semi-prep HPLC (method B) to afford an orange solid (1.2 mg, 100%). HRMS (ESI-TOF) *m/z* calcd for $C_{35}H_{48}BF_2N_6O_5^+$ $[MH]^+$ 681.3742; found; 681.3732. Analytical RP-HPLC; t_R = 18.12 mins, purity = 99%

6-((4,4-Difluoro-5,7-dimethyl-4-bora-3a,4a-diaza-s-indacene-3-propionyl)amido)-((2*S*)-1-((2-((2-((3-hydroxy-4-((7-methyl-2,3-dihydro-1*H*-inden-4-yl)oxy)butan-2-yl)amino)ethyl)amino)-2-oxoethyl)amino)-1-oxopropan-2-yl)hexanamide (8c).

The title compound was prepared according to the procedure described in general method B using reagents in the following quantities: (±)-((2*S*)-1-((2-((2-((3-hydroxy-4-((7-methyl-2,3-dihydro-1*H*-inden-4-yl)oxy)butan-2-yl)amino)ethyl)amino)-2-oxoethyl)amino)-1-oxopropan-2-yl)amine ditrifluoroacetate (**7a**) (1.3 mg, 1.99 μmol, 2.0 eq), BODIPY-FL-X-SE (0.5 mg, 9.95 μmol), DIPEA (0.6 mg, 4.98 μmol, 5.0 eq), and DMF (1.0 mL). Purification by semi-prep HPLC (method B) to afford an orange solid (0.77 mg, 97%). HRMS (ESI-TOF) *m/z* calcd for $C_{41}H_{60}BF_2N_7O_6^+$ $[MH]^+$; 794.4582 found; 794.4514. Analytical RP-HPLC; t_R = 16.10 mins, purity = 99%

(±)-*N*-(3-((3-((2-((3-Hydroxy-4-((7-methyl-2,3-dihydro-1*H*-inden-4-yl)oxy)butan-2-yl)amino)ethyl)amino)-3-oxopropyl)amino)-3-oxopropyl)-6-((4-(2-(4,4-difluoro-4,4a-dihydro-5-(thiophen-2-yl)-4-bora-3a,4a-diaza-s-indacene-3-yl)vinyl)phenoxy)acetamido)-hexanamide (9a).

The title compound was prepared according to the procedure described in general method B using reagents in the following quantities: (±)-(3-((3-((2-((3-hydroxy-4-((7-methyl-2,3-dihydro-1*H*-inden-4-yl)oxy)butan-2-yl)amino)ethyl)amino)-3-oxopropyl)amino)-3-oxopropyl)amine ditrifluoroacetate (**7b**) (2.0 mg, 3.0 μmol, 2.0 eq), BODIPY-630/650-X-SE (1.0 mg, 1.5 μmol, 1.0 eq) DIPEA (1.3 μL, 7.6 μmol, 5.0 eq) and DMF (1.0 mL). Purification by semi-prep HPLC (method B) to afford a blue solid (1.5 mg, 100%). HRMS (ESI-TOF) *m/z* calcd for $C_{51}H_{62}BF_2N_7O_7SNa^+$ $[MH+Na]^+$; 988.4390 found; 988.4373. Analytical RP-HPLC; t_R = 18.72 mins, purity = 99%

((±)-*N*-(3-((3-((2-((3-Hydroxy-4-((7-methyl-2,3-dihydro-1*H*-inden-4-yl)oxy)butan-2-yl)amino)ethyl)amino)-3-oxopropyl)amino)-3-oxopropyl)-4,4-difluoro-5,7-dimethyl-4-bora-3a,4a-diaza-s-indacene- 3-propionamide (9b).

The title compound was prepared according to the procedure described in general method B using reagents in the following quantities: (±)-(3-((3-((2-((3-hydroxy-4-((7-methyl-2,3-dihydro-

1*H*-inden-4-yl)oxy)butan-2-yl)amino)ethyl)amino)-3-oxopropyl)amino)-3-oxopropyl)amine ditrifluoroacetate (**7b**) (1.6 mg, 2.6 µmol, 2.0 eq), BODIPY-FL-SE (0.5 mg, 1.3 µmol, 1.0 eq) DIPEA (1.1 µL, 6.4 µmol, 5.0 eq) and DMF (1.0 mL). Purification by semi-prep HPLC (method B) to afford an orange solid (0.9 mg, 100%). HRMS (ESI-TOF) *m/z* calcd for $C_{36}H_{50}BF_2N_6O_5^+$ $[MH]^+$; 695.3898 found; 695.3885. Analytical RP-HPLC; t_R = 18.93 mins, purity = 99%

6-((4,4-Difluoro-5,7-dimethyl-4-bora-3a,4a-diaza-s-indacene-3-propionyl)amido)-*N*-(3-((3-((2-((3-hydroxy-4-((7-methyl-2,3-dihydro-1*H*-inden-4-yl)oxy)butan-2-yl)amino)ethyl)amino)-3-oxopropyl)amino)-3-oxopropyl)hexanamide (9c).

The title compound was prepared according to the procedure described in general method B using reagents in the following quantities: (±)-(3-((3-((2-((3-hydroxy-4-((7-methyl-2,3-dihydro-1*H*-inden-4-yl)oxy)butan-2-yl)amino)ethyl)amino)-3-oxopropyl)amino)-3-oxopropyl)amine ditrifluoroacetate (**7b**) (1.3 mg, 2.0 µmol, 2.0 eq), BODIPY-FL-X-SE (0.5 mg, 1.0 µmol, 1.0 eq) DIPEA (0.9 µL, 5.0 µmol, 5.0 eq) and DMF (1.0 mL). Purification by semi-prep HPLC (method B) to afford an orange solid (0.9 mg, 100%). HRMS (ESI-TOF) *m/z* calcd for $C_{42}H_{62}BF_2N_7O_6^+$ $[MH]^+$; 808.4739 found 808.4762. Analytical RP-HPLC; t_R = 16.64 mins, purity = 99%

(±)-*N*-(2-(2-(2-((3-Hydroxy-4-((7-methyl-2,3-dihydro-1*H*-inden-4-yl)oxy)butan-2-yl)amino)ethoxy)ethoxy)ethyl)-6-((4-(2-(4,4-difluoro-4,4a-dihydro-5-(thiophen-2-yl)-4-bora-3a,4a-diaza-s-indacene-3-yl)vinyl)phenoxy)acetamido)-hexanamide (12a).

The title compound was prepared according to the procedure described in general method B using reagents in the following quantities: (±)-3-((2-(2-(2-aminoethoxy)ethoxy)ethyl)amino)-1-((7-methyl-2,3-dihydro-1*H*-inden-4-yl)oxy)butan-2-ol dihydrochloride (**11**) (1.8 mg, 3.0 µmol, 2.0 eq), BODIPY-630/650-X-SE (1.0 mg, 1.5 µmol, 1.0 eq) DIPEA (1.3 µL, 7.6 µmol, 5.0 eq) and DMF (1.0 mL). The crude product was purified by pTLC (5:95 MeOH/DCM) to afford a blue solid (1.1 mg, 79%). HRMS (ESI-TOF) *m/z* calcd for $C_{49}H_{61}BF_2N_7O_7S^+$ $[MH]^+$; 952.4409 found; 952.4417. Analytical RP-HPLC; t_R = 21.90 mins, purity = 98%

(±)-*N*-(2-(2-(2-((3-Hydroxy-4-((7-methyl-2,3-dihydro-1*H*-inden-4-yl)oxy)butan-2-yl)amino)ethoxy)ethoxy)ethyl)-4,4-difluoro-5,7-dimethyl-4-bora-3a,4a-diaza-s-indacene-3-propionamide (12b).

The title compound was prepared according to the procedure described in general method B using reagents in the following quantities: (±)-3-((2-(2-(2-aminoethoxy)ethoxy)ethyl)amino)-1-((7-methyl-2,3-dihydro-1*H*-inden-4-yl)oxy)butan-2-ol dihydrochloride (**11**) (1.5 mg, 2.6 μmol, 2.0 eq), BODIPY-FL-SE (0.5 mg, 1.3 μmol, 1.0 eq) DIPEA (1.1 μL, 6.4 μmol, 5.0 eq) and DMF (1.0 mL). Purification by semi-prep HPLC (method B) to afford an orange solid (1.0 mg, 100%). HRMS (ESI-TOF) *m/z* calcd for $C_{34}H_{48}BF_2N_4O_5^+$ $[MH]^+$; 641.3680 found; 641.3697. Analytical RP-HPLC; t_R = 18.23 mins, purity = 97%

6-((4,4-Difluoro-5,7-dimethyl-4-bora-3a,4a-diaza-s-indacene-3-propionyl)amido)-*N*-(2-(2-(2-((3-hydroxy-4-((7-methyl-2,3-dihydro-1*H*-inden-4-yl)oxy)butan-2-yl)amino)ethoxy)ethoxy)ethyl)hexanamide (12c).

The title compound was prepared according to the procedure described in general method B using reagents in the following quantities: (±)-3-((2-(2-(2-aminoethoxy)ethoxy)ethyl)amino)-1-((7-methyl-2,3-dihydro-1*H*-inden-4-yl)oxy)butan-2-ol dihydrochloride (**11**) (1.2 mg, 2.0 μmol, 2.0 eq), BODIPY-FL-X-SE (0.5 mg, 1.0 μmol, 1.0 eq) DIPEA (0.9 μL, 5.0 μmol, 5.0 eq) and DMF (1.0 mL). Purification by semi-prep HPLC (method B) to afford an orange solid (0.8 mg, 100%). HRMS (ESI-TOF) *m/z* calcd for $C_{40}H_{59}BF_2N_5O_6^+$ $[MH]^+$; 754.4521 found; 754.4436. Analytical RP-HPLC; t_R = 18.51 mins, purity = 98%.

Supplementary Figures.

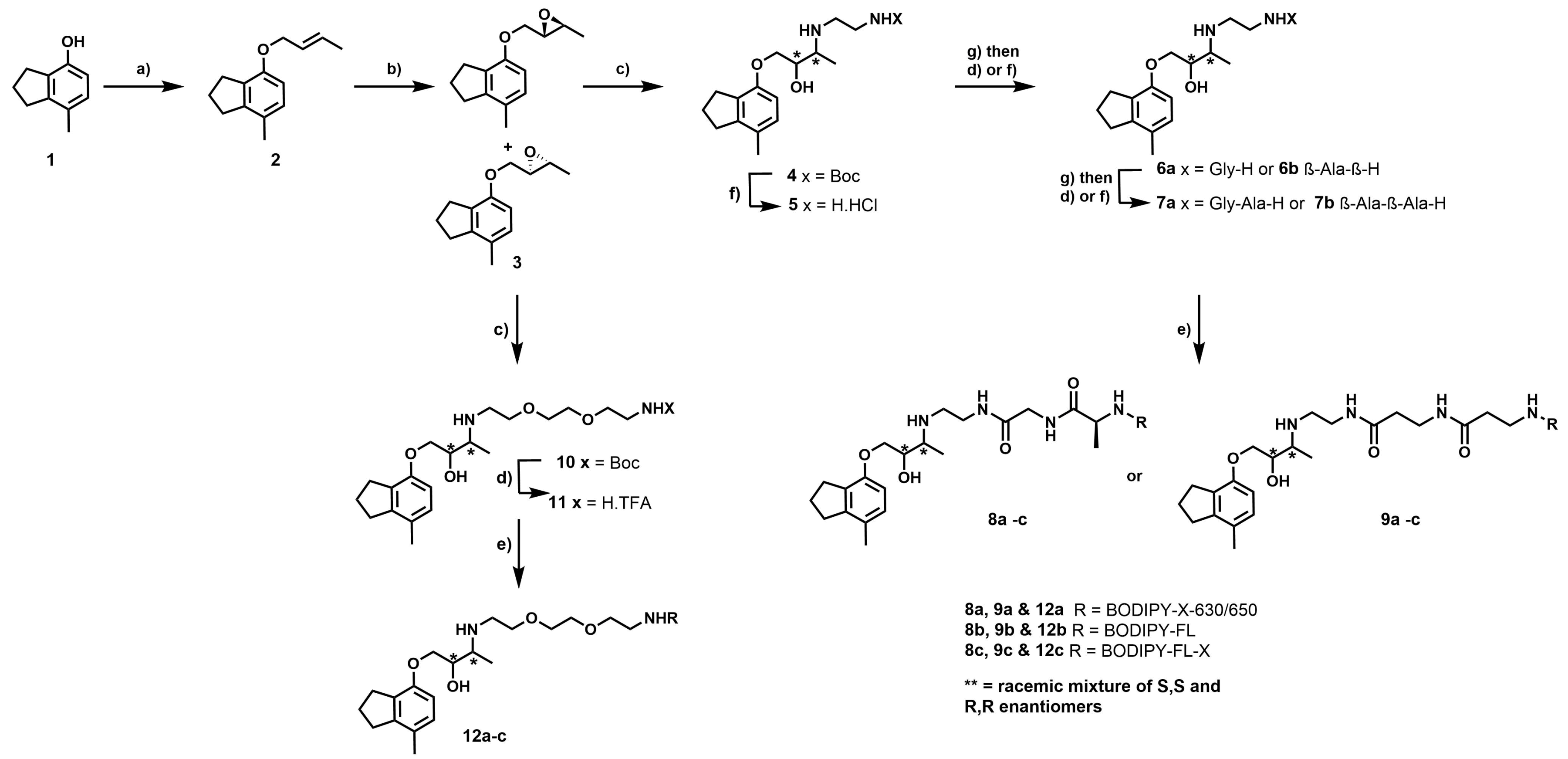

Supplementary Figure 1. Reaction Scheme for the synthesis of fluorescent ICI 118551 analogues. Reagents and Conditions**: a)** *trans*-crotyl alcohol, DIAD, PPh_3, THF, 0°C to rt, 6 days, 61% **b)** *m*-CPBA, DCM, 0°C to rt, 41% **c)** tert-butyl {2-[2-(2-aminoethoxy)ethoxy]ethyl}carbamate or *N*-Boc-ethylene diamine, MeOH, reflux, 3 days, 32% and 2 days, 67% respectively **d)** TFA/DCM (1:1), quantitative, **e)** BODIPY 630/650-X-SE, BODIPY-FL-SE or BODIPY-FL-X-SE, DIPEA, DMF, 79% to quantitative **f)** 4M HCl in dioxane/Et_2O (1:1), rt, 88% to quantitative **g)** selected amino acid, HBTU, DIPEA, DMF, rt, 53% – 65%.

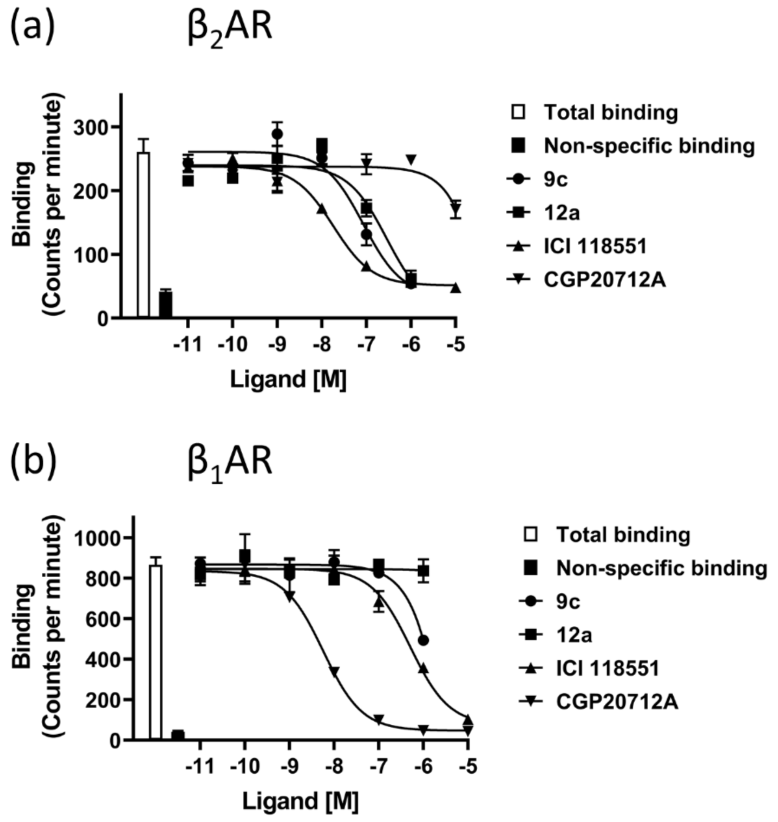

Supplementary Figure 2. Whole cell radioligand competition binding. Inhibition of the binding of ^{3}H-CGP12177 (0.75-1.2 nM) by **9c** (circle), **12a** (square) ICI 118,551 (upwards triangle) and CGP 20712A (downwards triangle) in CHO cells expressing (a) β_2AR or (b) β_1AR. Data represent mean ± SEM of triplicate determinations in a single experiment. Similar results were obtained in (a) 5 and (b) 6 further experiments (Table 3).

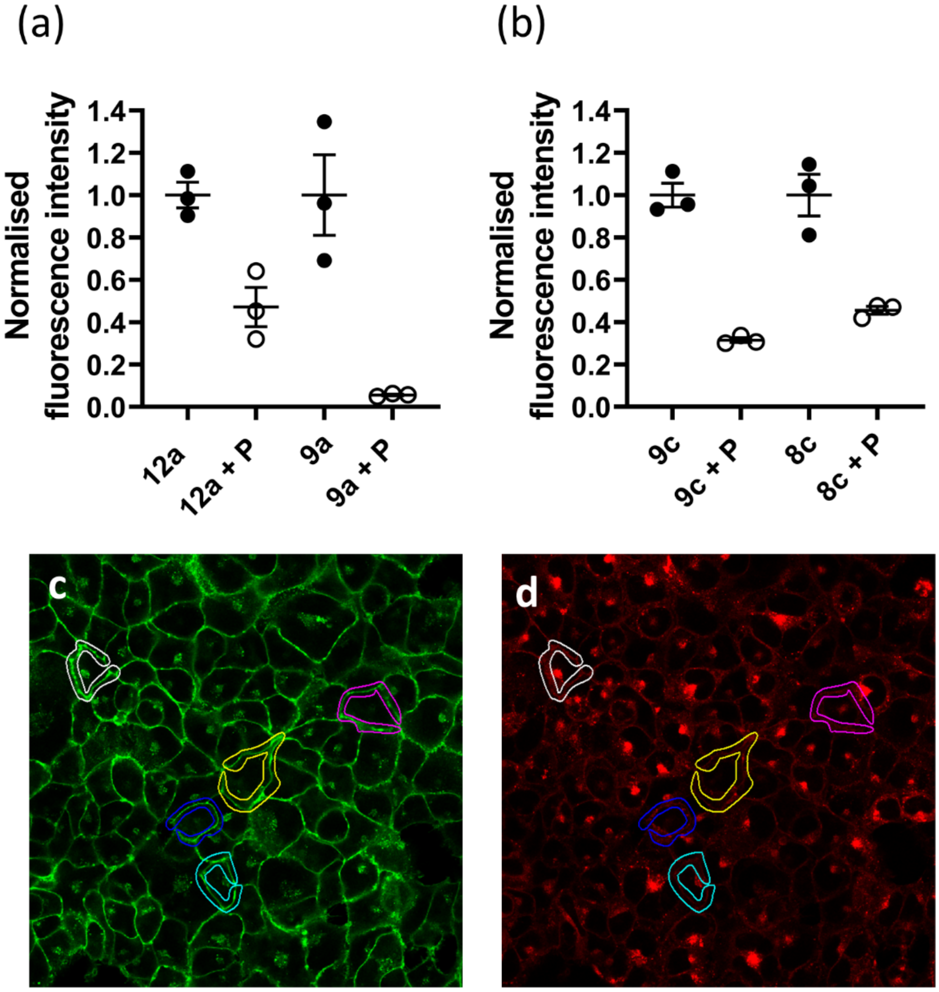

Supplementary Figure 3. Quantified fluorescence intensity of fluorescent ICI 118551 analogues. Mean fluorescence intensity was determined for 5 regions of interest (ROI) for 3 separate image captures for (a) **12a** and **9a** and (b) **9c** and **8c** in the absence (closed circles) or presence (open circles) of 10 µM propranolol (P). Fluorescence intensity was normalised to the mean intensity in the absence of propranolol. ROIs were detailed by hand on the (c) sSNAP channel capture and raw fluorescence intensity was measured in the (d) ligand channel. For illustration 5 ROIs on (c) sSNAPAF488 and the corresponding (d) 100 nM **12a** are shown. Gain and offset values were kept constant for each emission channel. Line and error bars represent mean ± S.E.M.

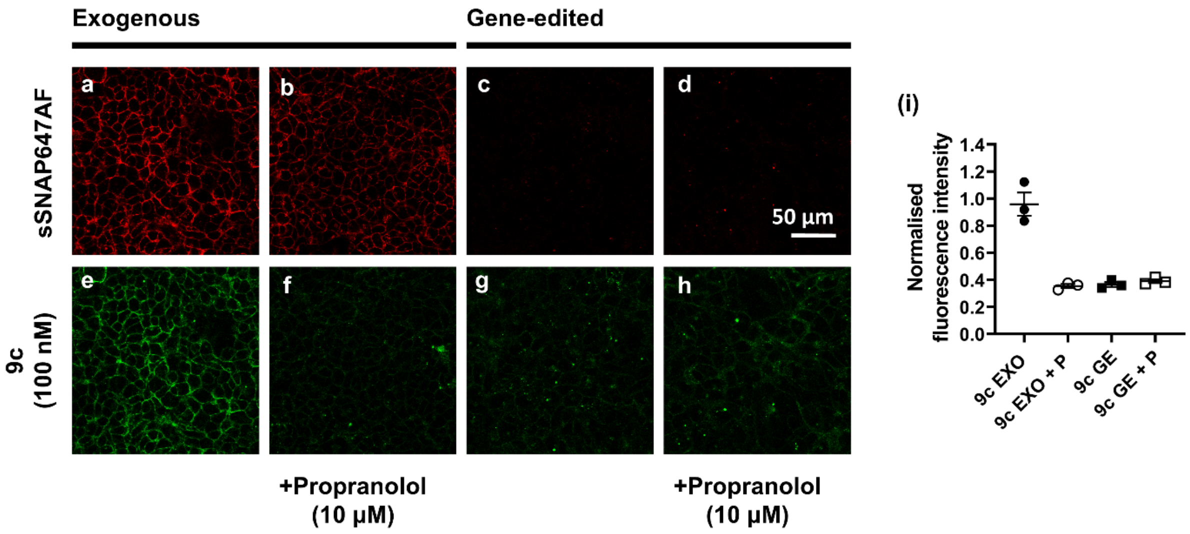

Supplementary Figure 4. Gene-edited β$_2$AR cannot be visualised through standard confocal microscopy. Hek293T cells either expressing the SNAP-β$_2$AR under exogenous (a-b, e-f) or endogenous promotion (c-d, g-h) were incubated for 30 min with 100 nM **9c** following 30 min pre-incubation with HBSS (a, e, c, g) or 10 µM propranolol (b, f, d, h). Any specific binding of **9c** on gene-edited lines (g) cannot be distinguished from non-specific binding displayed following propranolol incubation (f, h, i). (i) Mean fluorescence intensity due to **9c** was determined for 5 regions of interest (ROI) for 3 separate image captures on the exogenous (EXO, circles) and gene-edited (GE, squares) lines in the absence (closed) or presence (open) of 10 µM propranolol (P). Fluorescence intensity was normalised to the mean intensity in the absence of propranolol. Before ligand treatment the receptor was labelled with sSNAP647AF (0.5 µM) to visualise receptor (a-d) however due to low signal to noise the SNAP label can also not be detected in gene-edited lines (c-d). Gain and offset values were kept constant for each emission channel.
